# Recovery of the cortical chloroplast layer in the green alga *Chara* after local irradiation

**DOI:** 10.3389/fpls.2025.1544999

**Published:** 2025-05-05

**Authors:** Marion C. Hoepflinger, Margit Höftberger, Aniela Sommer, Florian Hohenberger, Michael Schagerl, Ilse Foissner

**Affiliations:** ^1^ Department of Environment and Biodiversity, University of Salzburg, Salzburg, Austria; ^2^ Department of Functional and Evolutionary Ecology, Faculty of Life Sciences, University of Vienna, Vienna, Austria

**Keywords:** actin cytoskeleton, characean internodal cells, chloroplast anchorage, CaCHUP1 (*Chara australis* chloroplast unusual positioning 1), confocal laser scanning microscopy, cortex regeneration, PAM fluorescence

## Abstract

The cytoplasm of characean internodal cells is characterized by a stationary layer of cortical chloroplast files and a mobile endoplasm moving along subcortical actin bundles. Occasionally, chloroplasts detach from the cortex and are passively carried along with the endoplasmic flow. Previous studies revealed that local irradiation with intense light causes chloroplast bleaching followed by a release into the endoplasm (“window formation”). We found that endoplasmic chloroplasts of *Chara australis* resettle at the window and align parallel to the streaming direction. The process takes several weeks with neither chloroplast division nor growth of proplastids being involved. Both release and re-attachment are actin-dependent. Resettled chloroplasts showed slightly, but significantly lower maximum quantum efficiency (Fv/Fm) values as compared with control regions. Extremely low Fv/Fm values were measured in chloroplasts at the border of the window even after three months indicating longevity, although with serious damage. In higher plants, a protein complex is responsible for the motility and anchorage of chloroplasts, with CHUP1 (CHLOROPLAST UNUSUAL POSITIONING 1) being an essential part. We discovered a homologous form CaCHUP1, encoding a polypeptide of 1201 amino acids with a calculated molecular mass of about 130 kDa. When transiently expressed in epidermal cells of *Nicotiana benthamiana* leaves, fluorescently tagged CaCHUP1 localizes to chloroplasts. We assume that CaCHUP1 is involved in the anchorage of chloroplasts and in the polymerization of actin filaments, but not in active movement. Our study revealed that endoplasmic chloroplasts can re-anchor at the cell cortex thereby refilling chloroplast-free regions, which we interpret as a repair mechanism after various kinds of damage. It confirms that chloroplasts use different strategies for repositioning, either via polymerization of cp-actin or via cytoplasmic streaming.

## 1 Introduction

In higher plants, ferns, mosses and sessile algae, chloroplasts relocate towards illuminated areas within the cell (accumulation response), or to shaded regions if exposed to excess light (avoidance response; e. g. Wada and Kong, 2018). Chloroplast movement thereby ensures efficient photosynthesis under various light conditions and prevents photodamage (e.g. Kasahara et al., 2002). With few exceptions, both accumulation and avoidance responses depend on the actin cytoskeleton and associated proteins (Kong et al., 2024; Oikawa et al., 2003, 2008; Suetsugu and Wada, 2016; Suetsugu et al., 2010, 2017; Wada and Kong, 2018, 2019; Yamamoto-Negi et al., 2024). Actin-dependent chloroplast movements have also been described for cold-treated (Kimura and Kodama, 2016) and for injured cells (Gamalei et al., 1994).

The chloroplasts of characean algae share properties with those of vascular plants to which they are closely related (Delwiche and Cooper, 2015; Domozych and Bagdan, 2022; McCourt et al., 2017; Turmel et al., 2013 for review). However, they are immobilized at the cell periphery and are unable to relocate actively in response to changing light or temperature via polymerization of actin filaments. Stromules (Natesan et al., 2009) have also not yet been described in characean algae.

**Figure 1:**
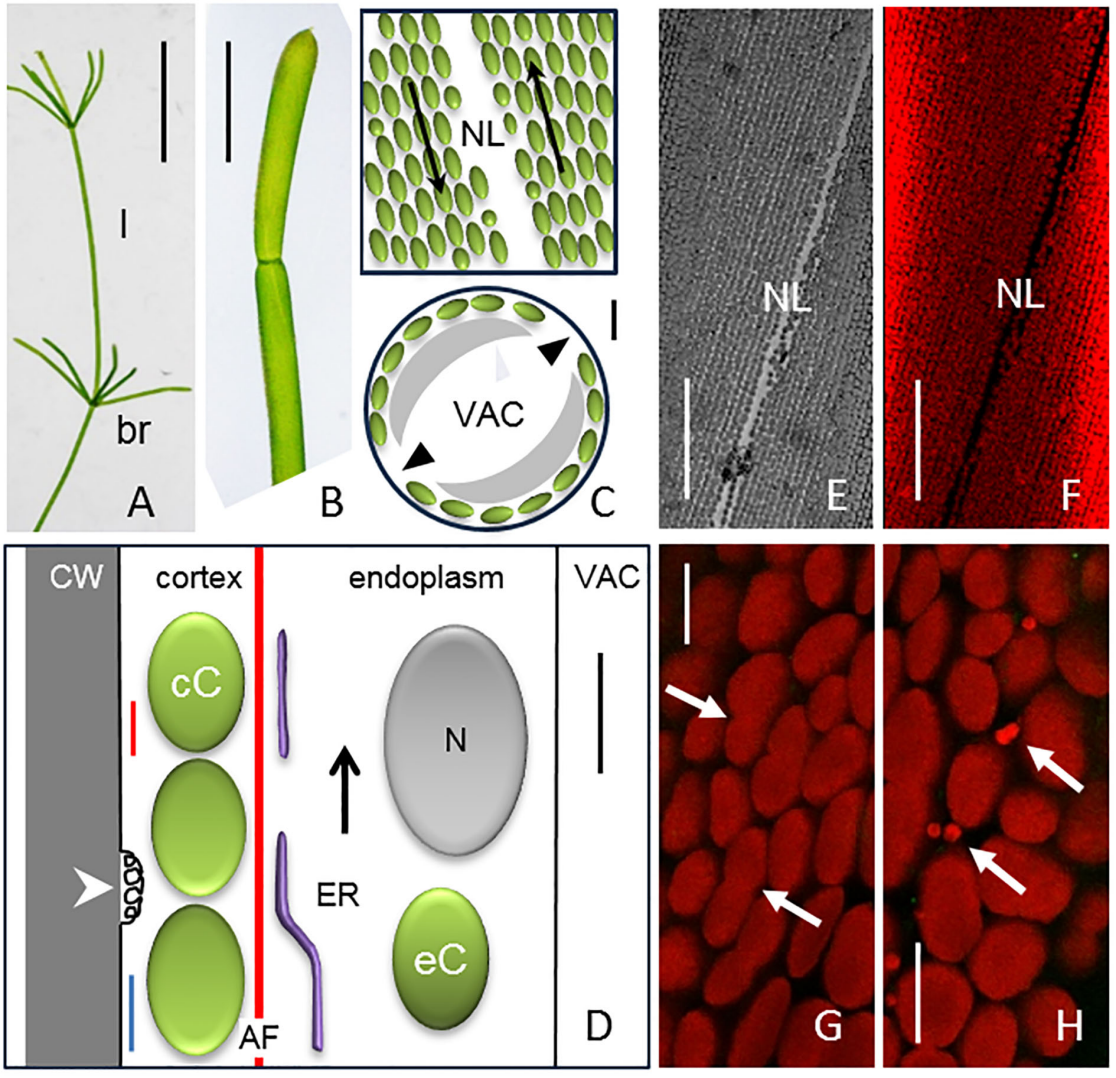
Thallus, organization of internodal cell and cortical chloroplasts. **(A)** Thallus of *Chara australis* (I = internodal cell of main axis, br = branchlet). **(B)** Distal and part of proximal branchlet internodal cell. **(C, D)** Schematic drawings of internodal cell organization (compare Foissner and Wasteneys, 2012). **(C)** Stationary chloroplasts (green) are arranged in helical files (tangential view, upper image). Opposite streams of endoplasm (arrows) are separated by the neutral line (NL and arrow heads in the cross-section; lower image). The grey areas in the cross section represent the endoplasm; VAC = central vacuole. **(D)** Fine structure of internodal cell (longitudinal section). The cortex mainly consists of stationary (cortical) chloroplasts (cC). Actin filaments (thin red line) and microtubules (blue line) are located at the plasma membrane which may by convoluted to charasomes (arrow head). Subcortical actin bundles (AF; thick red line) are attached to the inner side of the chloroplasts and generate endoplasmic streaming by interaction with myosin coated endoplasmic reticulum (ER). The endoplasm contains nuclei (N), endoplasmic chloroplasts (eC) and various other organelles (not shown). VAC = central vacuole. **(E–H)** Bright field image **(E)** and fluorescence images [**(F–H)**; single optical sections] of chloroplasts in a mature, non-elongating branchlet cell. Note dumbbell-shaped chloroplasts [arrows in **(G)**] and tiny organelles [arrows in **(H)**] in the higher magnifications. Bar = 1.5 cm **(A)**, 300 µm **(B)**, 5 µm **(C, D)**, 100 µm **(E, F)**, 10 µm **(G, H)**. Schematic images are only approximately drawn to scale.

We investigated the repair of the cortical chloroplast layer in the *Chara australis* R.Brown after strong local irradiation. The internodal cells of *C. australis* have a cylindrical shape and are up to several cm long (**Figures 1A, B**). The cells have been proved as a valuable experimental system to study various aspects of cell biology, including ion transport and electrophysiology (reviewed by e.g. Beilby and Bisson, 2012; Beilby, 2016; Beilby and Casanova, 2014), cell organization and organelle movements (reviewed by Foissner and Wasteneys, 2012, 2014). The immobilization of chloroplasts in the cortex allows to study interactions between chloroplasts, and between chloroplasts and the plasma membrane (e. g. Bulychev and Komarova, 2014; Bulychev, 2020). Although the genome of *Chara braunii* has been published (Nishiyama et al., 2018) and the subcellular proteome of *Chara australis* internodal cells has been analyzed (Pertl-Obermeyer et al., 2018), the molecular biology of the Characeae is still at its infancy (Domozych and Bagdan, 2022; Holzhausen et al., 2022; Kurtović et al., 2024).

The cytoplasm of *Chara* internodal cells is partitioned into a stationary cortex and a mobile endoplasm, which performs rotational streaming (**Figures 1C, D**; for references see Foissner and Wasteneys, 2014). The tangential (upper image) and the cross-section (lower image) in **Figure 1C** illustrate that chloroplasts are organized as files which have been shown to follow the helical strain pattern of the cell wall (Green, 1964). Up- and down-streaming endoplasm is separated by the neutral line (NL and arrow heads in **Figure 1C**). The cortex consists of files of immobile (cortical) chloroplasts (cC in **Figure 1D**); cortical actin filaments and microtubules are located at the plasma membrane which contains convoluted domains (“charasomes”) that play a role in ion transport (arrow head in **Figure 1D**; for references see Absolonova et al., 2018). Subcortical actin bundles consisting of up to 100 filaments are attached to the inner side of the cortical chloroplast and generate endoplasmic streaming by interaction with myosin-coated cisternae of the endoplasmic reticulum (ER; **Figure 1D**). The endoplasm also contains numerous nuclei (N), mitochondria, Golgi bodies, multivesicular bodies and other organelles. Cortical chloroplasts occasionally detach from the cortex and are then carried passively with endoplasmic flow (eC in **Figure 1D**). They may, however, perform active, myosin-dependent rotational movements, if actin bundles (rings) are attached to their surface (Jarosch, 1956; Wasteneys et al., 1996). Actin rings are occasionally present at chloroplasts anchored at the neutral line (Wasteneys et al., 1996). A huge vacuole (VAC) occupies the center of the cells.

Chloroplasts can be removed by various treatments, e. g. mechanical damage (Chen, 1983), chemicals which interfere with the Ca^2+^ metabolism (Foissner, 1988; 1990) and by local irradiation with strong light (Kamitsubo, 1972). The latter method was successfully used to study the regeneration of the actin and microtubule cytoskeleton in these chloroplast-free areas called “windows” (Williamson and Hurley, 1986; Williamson et al., 1984; Foissner and Wasteneys, 2012). The sequence of events that have been described so far during and after local irradiation are (1) the bleaching and rounding up of cortical chloroplasts, (2) the retardation or even arrest of endoplasmic streaming, (3) the dissolution of cortical actin filaments, microtubules and (possibly) subcortical actin bundles, (4) the detachment of bleached chloroplasts and their release into the streaming endoplasm (“window formation”). The recovery process includes (5) the regeneration of the subcortical actin bundles including recovery of active cytoplasmic streaming, and (6) the reappearance of microtubules except those of cortical actin filaments (see review by Foissner and Wasteneys, 2012). Longer irradiation induces the secretion of a wound wall, which requires a three-dimensional actin meshwork for the transport of wall-forming vesicles (Foissner and Wasteneys, 1997).

In the current study, we aimed to discover potential repair mechanisms in windows. We assumed either a refilling through dividing cortical chloroplasts or via a resettlement of endoplasmic chloroplasts. We also searched for an explanation at the molecular level, which could be e.g. the CHUP1 protein, which is required for photo-relocation of chloroplasts in vascular plants (Kong et al., 2024). The outcome of this study is also of relevance for plants living in their natural habitat. The organisms face various challenges in the environment; repair mechanisms of mechanical and radiation damage are indispensable for their survival.

## 2 Materials and methods

### 2.1 Algal material and culture conditions

Male thalli of *Chara australis* R.Brown were grown at room temperature in 10-50 L containers in a mixed substrate of soil, peat and sand covered by distilled water. Fluorescent lamps provided a photon flux density of 5 μmol·m^−2^·s^−1^ photosynthetically active radiation (PAR) at the water surface for 14 hours per day. The maximum age of cultures used for experiments was two months. Internodal cells of lateral branchlets (axis with limited growth) were isolated from the 2^nd^ or 3^rd^ node of the thallus and stored in artificial fresh water (AFW, 0.1 mM NaCl, 0.1 mM KCl, 1 mM CaCl_2_; unbuffered pH 5.6) until use.

### 2.2 Inhibitor treatments and staining

The involvement of the cytoskeleton in chloroplast detachment and resettlement was investigated using specific inhibitors. The actin inhibitor cytochalasin D reversibly arrests endoplasmic streaming in characean internodal cells and was used at concentrations of 10 and 20 µM. Oryzalin depolymerizes microtubules and paclitaxel inhibits the dynamics of microtubules and stabilizes microtubules. Both oryzalin and paclitaxel were applied at concentrations of 8 and 10 µM, respectively. Stock solutions of cytochalasin D (Sigma, 10 mM in DMSO), oryzalin (Riedel-de Haen, 10 mM in DMSO) and paclitaxel (Molecular probes, 2 mM in DMSO) were diluted with AFW; controls contained the equivalent concentration of DMSO. Inhibitor and control solutions were prepared freshly for short time experiments or were replaced every week for long term experiments.

Actin was labelled by perfusion of cells with Alexa Fluor 488 phalloidin (Invitrogen) as described in Foissner and Wasteneys (2007).The 6.6 µM stock solution in methanol was dissolved in perfusion solution (200 mM sucrose, 70 mM KCl, 4.49 mM MgCl_2_, 5 mM EGTA, 1.48 mM CaCl_2_, 10 mM Pipes, pH 7). Images were taken 5-20 min after dye loading.

### 2.3 Light microscopy and window formation

**Figure 2:**
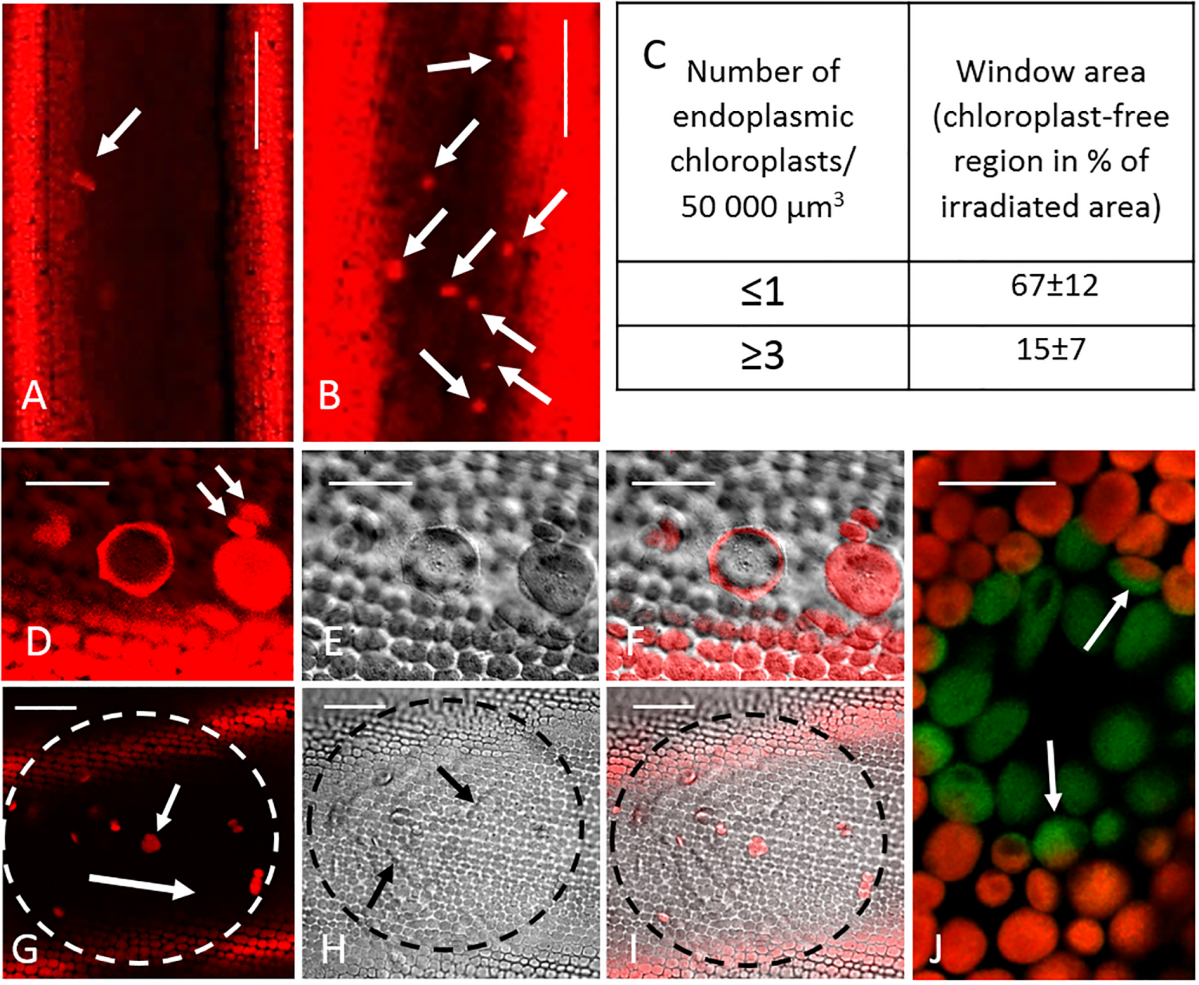
Endoplasmic chloroplasts and window formation. **(A, B)** Endoplasmic chloroplasts (arrows) in two cells. Note different abundance (0.3 and 3 chloroplasts/50000 µm^3^ endoplasm, respectively, for these images). **(C)** Recovery of the cortical chloroplast layer depends on the presence of endoplasmic chloroplasts. Cells were examined 4 weeks after irradiation and the remaining (non-healed) window area was calculated. Data are means ± SD, and are significantly different (t-test; P ≤ 0.005; 14 windows (cells) from 3 different thalli were investigated). **(D–F)** Endoplasmic chloroplasts at the neutral line. The left giant chloroplast is slightly cup-shaped and has a polygonal outline; the right giant chloroplast is disc-shaped. Endoplasmic chloroplasts with normal size are indicated by arrows. Cortical chloroplasts are seen at the lower area of the images. **(G–I)** Local irradiation (circled area) with sufficient energy and duration causes bleaching of cortical chloroplasts and disturbance of endoplasmic streaming. Images were taken 1 hour after irradiation. Note bright fluorescence of cortical chloroplasts outside the window and of moving endoplasmic chloroplasts located in the inner layer). The long arrow in G indicates the streaming direction, the short arrow points at an endoplasmic chloroplast. Arrows in H indicate bulges (uneven distribution of endoplasm beneath the window). **(J)** Bleached chloroplasts in a small window. Note partially bleached chloroplasts (arrows) at the border of the window. **(A, B, D, G, J)** fluorescence of chloroplasts); **(E, H)** bright field images, **(F, I)** merged images. All images are single optical sections. Bar = 100 µm **(A, B)** 40 µm **(G–I)**, 20 µm **(D–F)** and 10 µm **(J)**.

Chloroplast resettlement at windows depended on the presence of endoplasmic chloroplasts (compare **Figures 2A–C**). We therefore counted the relative number of these chloroplasts before irradiation using consecutive, non-overlapping images of the endoplasm and assuming an endoplasm thickness of 5 µm. Windows were produced in cells with at least 1 chloroplast per 50 000 µm^3^ endoplasm.

Windows with a roundish or rectangular shape and a size between 10 000 to 40 000 µm^2^ were produced by local irradiation with a 50 or 100 W mercury lamp or a halide lamp (highest intensity) guided through blue light emitting fluorescence filter cubes (emission 450-490 nm) and using 40x lenses (numerical apertures between 0.7 and 0.85) of various fluorescence microscopes. The light intensity at the surface of the cell was about 4000 μmol.m^−2^.s^−1^ PAR (MC-MQS/OVV Microscopy Quantum Sensor, Walz, Effeltrich, Germany). The irradiation time required to retard streaming and to induce the detachment of chloroplasts mainly depended on the light intensity of the respective lamp and on the growth status of cells. At the provided conditions and with the cells used, irradiation for 1.5-6.0 min was sufficient for release of cortical chloroplasts within one day.

Bright field and fluorescent images were obtained using either a Leica TCS SP5 confocal laser scanning microscope (CLSM) coupled to a DMI 6000B inverted microscope (Mannheim, Germany), or a Zeiss LSM 510 coupled to an Axiovert inverted microscope (Jena, Germany). The red chlorophyll fluorescence was detected between 637 and 750 nm (Leica; tunable detection) or beyond 585 nm using a long pass filter (Zeiss). The fluorescence of Alexa Fluor 488 phalloidin was recorded between 500 and 573 nm. Bright field images of chloroplasts were also taken using a digital color camera (Canon EOS) attached to a Leitz Dialux 20 compound microscope.

CLSM images shown in this study are either single optical sections or projections of Z-stacks processed using Leica or Zeiss software and GIMP (GNU Image Manipulation Program; https://www.gimp.org).

### 2.4 Electron microscopy

Branchlet internodal cells were chemically fixed with 1% glutaraldehyde for 20 min at room temperature, followed by post-fixation with 2% OsO_4_ for 12 h at 4°C. Samples were dehydrated with ethanol, then transferred to propylene oxide and embedded in Agar low viscosity resin (Agar Scientific, Rotherham, UK) (Foissner et al., 2016). Part of the samples were also high-pressure frozen and cryo-substituted as described in Lütz-Meindl and Aichinger (2004), followed by dehydration with ethanol, transfer to propylene oxide and embedding in LR Gold (London Resin, Reading, UK).

### 2.5 Pulse amplitude modulation fluorescence

To get insight into the photosynthetic performance of PSII in resettled chloroplasts and in control regions for a comparison, fluorescence yields were measured and visualized by means of an imaging PAM fluorescence device (Imaging PAM M‐Series, Microscopy version, Walz company, Effeltrich, Germany). We measured the maximum efficiency of PSII during low light of 20 µmol photons m^-2^. s^-1^ (Fv’/Fm’ LL) and high light of 60 µmol photons m^-2^. s^-1^ (Fv’/Fm’ HL). Additionally, maximum quantum efficiency of PSII (Fv/Fm) was measured after 5 min of dark acclimation of cells. Fv/Fm is defined as Fv/Fm =(Fm − F0)/Fm (Maxwell and Johnson, 2000) and provides information about the overall photosynthetic performance; a reduced Fv/Fm indicates different types of plant stress. Fm represents the maximum dark fluorescence after a strong flash of light (all PSII reduced), Fm’ is the maximum fluorescence in ambient light. F0 is the minimum fluorescence in the dark (all PSII oxidized), F0’ the minimum fluorescence immediately after switching off ambient light; Fv is called variable fluorescence during darkness, Fv’ variable fluorescence during light exposure. We compared the development of photosynthetic performance of various regions over several weeks: upstream and downstream regions of the window (controls), borders of the window and the window itself.

### 2.6 Statistical analysis

Chloroplast numbers and areas were calculated using ImageJ (https://imagej.nih.gov). Diagrams were produced using Sigma Plot V14.5 (https://systatsoftware.com). Mann-Whitney-U tests were calculated to test significant effects of inhibitors oryzalin and paclitaxel. For cytochalasin D, a general linear mixed model (GLMM) was applied with window area taken as dependent variable, time and treatment set as fixed factors, and the replicates as fixed random factors. T-test was used after the normal distribution was checked with Shapiro-Wilk test. Data were obtained from different cells collected from at least 3 thalli.

PAM fluorescence data were analyzed with GLMM. Yield was taken as dependent variable, time and area were set as fixed factors, and the replicates as fixed random factors. For each region, 16 cells (replicates) from whorls of 5 thalli were repeatedly measured at the start of the experiment, after 1, 2, 3, 7, 10, 17, 29 and 79 days (end of the experiment). Statistics were performed with the jamovi package V2.3.28.0 (https://www.jamovi.org), jmw and GAMLj suite (https://gamlj.github.io/) installed.

### 2.7 Cloning and protein analyses

*Arabidopsis* CHUP1 protein (GenBank accession number: OAP01771) was used as a template to screen for homologous sequences in the transcriptomic database of *Chara australis* described in Pertl-Obermeyer et al. (2018). To verify the obtained CHUP1-like open reading frame from CL5265.Contig1_All, fresh total RNA from *C. australis* thalli was extracted using TRI-Reagent according to the manufacturer instructions (Sigma-Aldrich - Merck, Darmstadt, Germany). Residual genomic DNA was digested by RNase-free DNase (EN0521, Thermo Fischer Scientific, Waltham, MA, USA) and first-strand cDNA was synthesized from 1 μg total RNA by M-MuLV Reverse Transcriptase (RevertAid; EP0441, Thermo Fischer Scientific) using an anchored oligo(d)T primer-mix according to the supplier’s protocol. The obtained cDNA was used as a template for PCR amplification. All PCRs were performed with Phusion High-Fidelity DNA polymerase (F530S, Thermo Fischer Scientific) according to the manufacturer’s instructions and verified by sequencing. CaCHUP1 was cloned using the primers CL5265_fwd (5’-ATGTGGGTTCGATTCGCCCTCA-3’) and CL5265_rev (5’-CTACTGCTTGGCGGCGGGAGATT-3’). The amplicon (3606 base pairs) was sub-cloned into pJet1.2 cloning vector (Thermo Fischer Scientific, #K1231). The sequence vas verified by sequencing and named *Chara australis* CHLOROPLAST UNUSUAL POSITIONING 1 (CaCHUP1). The expression vector *pGI-AtUBQ10p::CaCHUP1::GFP* was created using the Hot-Fusion protocol as described in (Fu et al., 2014). This process involved our *pGI-AtUBQ10p::GFP* vector (described in Hoepflinger et al., 2013), the primers AtUBQ10p_CaCHUP1_HF_F (5’-AGGTCGACGGTATCGATAATGTGGGTTCGATTCGCCCTCA-3’) and CaCHUP1_GFP_HF_R (5’-TTCTCCTTTACTCATCCCCTGCTTGGCGGCGGGAGATT-3’) as well as the restriction enzyme sites SmaI and HindIII. The resulting expression vector *pGI-AtUBQ10p::CaCHUP1::GFP* contained an *A. thaliana* ubiquitin-10 promoter (AtUBQ10p; At4g05320), CaCHUP1 and a C-terminally fused *GFP*. This vector was transformed into *Agrobacterium tumefaciens* strain GV3101 (harboring the pSoup helper plasmid) and transiently expressed in *Nicotiana benthamiana* leaves as described in detail in Hoepflinger et al. (2013). Briefly, four-weeks old *N. benthamiana* plants, grown on standard fertilized soil (type ED73) in a growth chamber (16/8 h light/dark cycle at 22°C to 23°C, and a relative humidity of 60%), were used for *Agrobacterium*-mediated transient protein expression. *A. tumefaciens* carrying either *CaCHUP1::GFP* or a P19 repressor were grown to late-exponential phase (about 16 h) in liquid YEB medium. Cultures were harvested by centrifugation at room temperature (6000g, 5 min) and the resulting pellets were resuspended in infiltration buffer [10 mM MES/KOH (pH 5.6), 10 mM MgCl_2_, 150 μM acetosyringone]. Bacterial concentration was determined photometrically and both suspensions were combined to a final concentration of OD_600_ = 0.5 for *A. tumefaciens* carrying *CaCHUP1::GFP*, and OD_600_ = 0.25 for *A. tumefaciens* carrying the P19 repressor. After 2 h of incubation at room temperature, *N. benthamiana* leaves were infiltrated with the described suspension using a 1 ml syringe without needle. Tobacco plants were replaced to the growth chamber and examined after 3 to 4 days of expression. All obtained sequences and constructs were verified by sequencing. CaCHUP1 GenBank accession number PQ306591.

Clustal Omega (EMBL-EBI) was used for protein sequence alignments and conserved domains were detected using InterPro (Mitchell et al., 2014). The following sequences were compared: Charophytes: *Chara australis* CHUP1 (this paper), *Chara braunii* hypothetical protein CBR_g4598 (GenBank: GBG69767), *Klebsormidium nitens* CHUP1B (GenBank: GAQ78600); liverwort: *Marchantia paleacea* hypothetical protein Mapa_014704 (GenBank: KAG6543864); moss: *Physcomitrium patens* CHUP1 isoform X1 (NCBI Reference Sequence: XP_024379808); fern: *Adiantum capillus-veneris* CHUP1A (GenBank: BAG54845); eudicot: *Arabidopsis thaliana* CHUP1 (GenBank: OAP01771); Amino acid sequence identities were calculated with the bioinformatic software CloneManager (http://www.scied.com/).

For western blot analysis, thalli of *C. australis* were homogenized in liquid nitrogen using mortar and pestle. 2.5 volumes of ice cooled extraction buffer (100 mM NaH_2_PO_4_ (pH 7.8), 100 mM KCl, 1 mM DTT, 0.1% Tween) were added and the suspension was shaken for 15 min on ice followed by centrifugation (15 min, 7500 × g, 4°C). The supernatant was separated on a 10% acrylamide separation gel. Proteins were blotted onto a polyvinylidene difluoride membrane (PVDF; Merck, Darmstadt, Germany) for 70 min at 100 V. The membrane was blocked for 1.5 h in TBST-BSA (1% BSA, w/v) at room temperature followed by 1.25 h in TBST-BSA with 1:1000 dilution of α-CaCHUP1 antibody (affinity purified antiserum derived from rabbit, 0.5 µg/ml from Davids Biotechnology GmbH, Regensburg, Germany). The PVDF membrane was washed three times for 5 min each in TBST. The secondary antibody, a monoclonal anti-rabbit IgG coupled to alkaline phosphatase (Sigma-Aldrich), was used at a concentration of 1:20,000 in TBST for 1 h. After another wash step (3 × 5 min in TBST), protein labelling was detected using Tropix^®^ CDP-Star (T2146, Thermo Fischer Scientific) and the LAS 3000 mini-imaging system (Fujifilm, Tokyo, Japan).

## 3 Results

### 3.1 Cortical and endoplasmic chloroplasts

**Figure 1** shows a thallus of *Chara australis* (**Figure 1A**), two adjacent internodal cells of a branchlet (**Figure 1B**), and schematic images of the organization of internodal cells (**Figures 1C, D**; see introduction for details).

Bright field and fluorescence images of cortical chloroplast files in a fully grown branchlet cell are shown in **Figures 1E, F**. Most chloroplasts had an ovate to elongate shape with lengths ranging between 5 and 11 µm, but dumbbell-shaped chloroplasts or chloroplasts with division furrows smaller roundish organelles were also present (**Figure 1G**, arrows). Dot-like organelles (diameter up to 2.4 µm) with typical chlorophyll autofluorescence, are also seen (**Figure 1H**, arrows).

Chloroplasts were occasionally released from the cortex and moved passively along with endoplasmic flow, which was up to 100 µm s^-1^ in our experiments (**Supplementary Video 1**). Their abundance varied considerably (**Figures 2A, B**). Our previous observations indicated that the presence of endoplasmic chloroplasts is a prerequisite for the resettlement of the chloroplast layer at windows (**Figure 2C**). Most endoplasmic chloroplasts were comparable in size and shape to those found in the cortex, but significantly larger, disk-like or broadly ellipsoidal giant chloroplasts with diameters up to 32 µm (**Figures 2D–F**) and chloroplasts with division furrows could also be observed. Within the streaming endoplasm, many chloroplasts additionally performed active rotational movement (**Supplementary Video 2**) due to the presence of actin rings at their surface (see below).

### 3.2 Release of chloroplasts after local irradiation with intense blue light

Cells were irradiated with intense blue light until the endoplasm became unevenly distributed (**Figures 2G–I**) due to local retardation; sometimes active streaming even stopped. The endoplasm then moved passively and with slower velocities through the irradiated area due to delivery from undisturbed regions (**Supplementary Video 3**; **Supplementary Figures 1A-C**). The irradiated, bleached cortical chloroplasts (**Figure 2J**) became roundish, performed wiggling or trembling motions (**Supplementary Video 4**), detached from the cell periphery, and were removed with the endoplasm during the following hours (**Supplementary Figures 2A, B**). They were mostly released in groups, which remained visible up to several weeks after window formation, without signs of further degradation. Chloroplasts at the border of the window were sometimes partly bleached (arrows in **Figure 2J**). During irradiation, the red chlorophyll fluorescence decreased, and the green fluorescence of chloroplasts became distinct.

**Figure 3:**
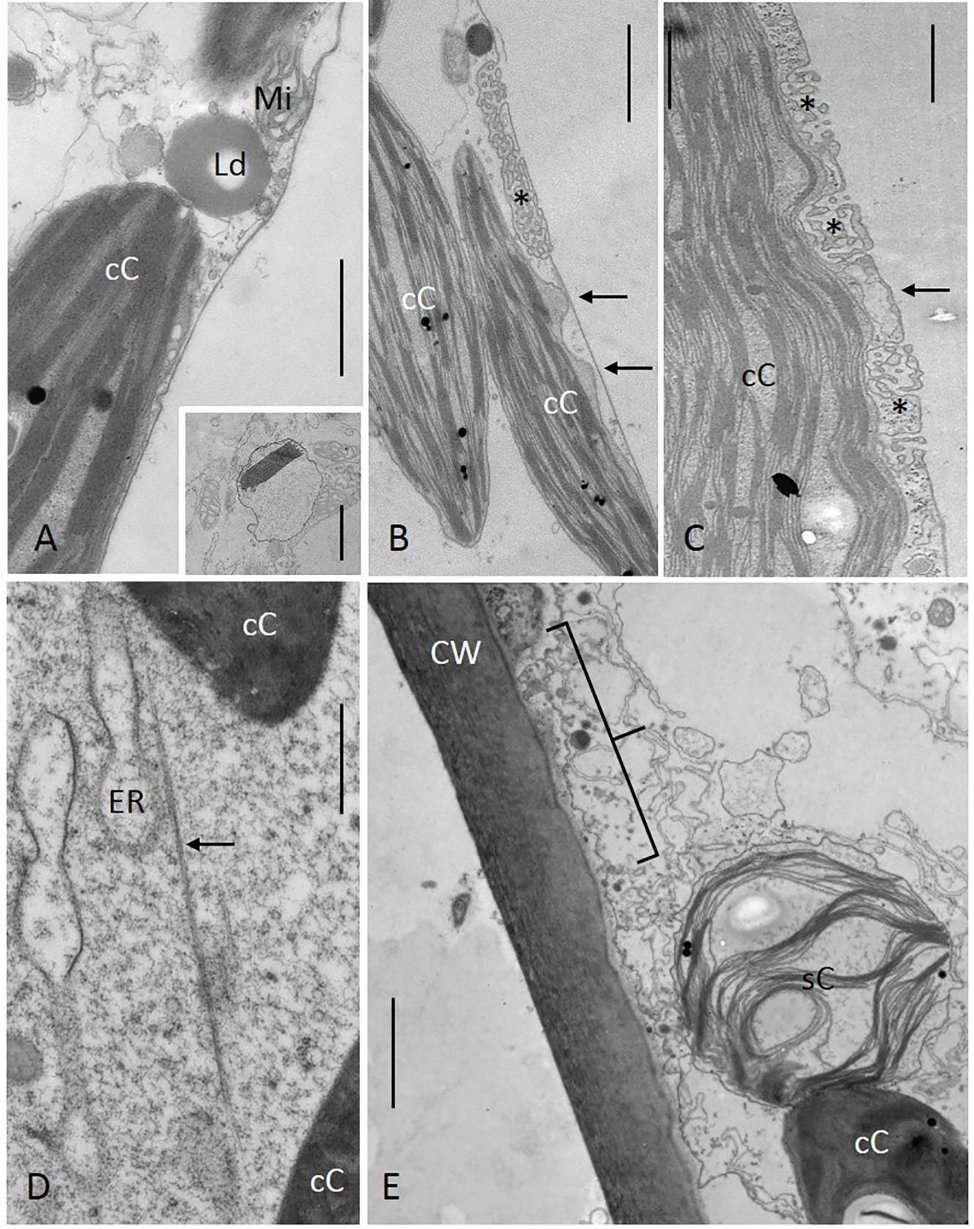
Fine structure of branchlet internodal cells. **(A–D)** Control regions, **(E)** window. The outer membrane of the cortical chloroplast (cC) is located adjacent to the plasma membrane **(A, B)**, to the inner surface of charasomes [asterisks in **(B, C)**] and to cisternae of the endoplasmic reticulum [arrow in **(C)**]. **(B)** is a tangential section of cortical chloroplasts with lobes contacting the plasma membrane (arrows). The inset in **(A)** is a section through a proplastid. **(D)** shows a subcortical actin bundle (arrow) spanning the region between two chloroplasts. Note cisternae of the endoplasmic reticulum (ER). **(E)** Cross-section through the border of a window. The swollen chloroplast (sC) was probably about to detach (bracket in E indicates part of the window). Detachment of the plasma membrane from the cell wall is a fixation artefact. **(A-B)**, **(E)** Chemical fixation, **(D)** cryofixation. CW cell wall, Ld lipid droplet, Mi mitochondrium. Bar = 2 µm **(E)**, 1 µm [**(A–C)**, inset in A] and 500 nm **(D)**.

Electron micrographs show chloroplasts in the control regions (**Figures 3A-C**) and at the border of a window (**Figure 3D**). In control regions, the chloroplast surface is closely attached to the plasma membrane (**Figure 3A**), but not always along its whole length. Occasionally, the putative attachment or at least the contact sites are zipped out (arrows in **Figure 3B**). Close contact is also observed between chloroplasts and charasomes (convoluted domains of the plasma membrane; asterisks in **Figures 3B, C**), and between chloroplasts and cisternae of the cortical ER (arrow in **Figure 3C**). The regions between the contact sites are occupied by mitochondria and other organelles (**Figures 3A–C**). The inset in **Figure 3A** shows a proplastid which probably corresponds to the small autofluorescent organelles visible in the CLSM (compare **Figure 1H**). A subcortical actin filament bundle is illustrated in **Figure 3D** (arrow). Its interaction with ER cisternae generates streaming. **Figure 3E** shows part of a newly formed window one hour after local irradiation. A swollen, detaching chloroplast (sC) is seen at the border of the window.

**Figure 4:**
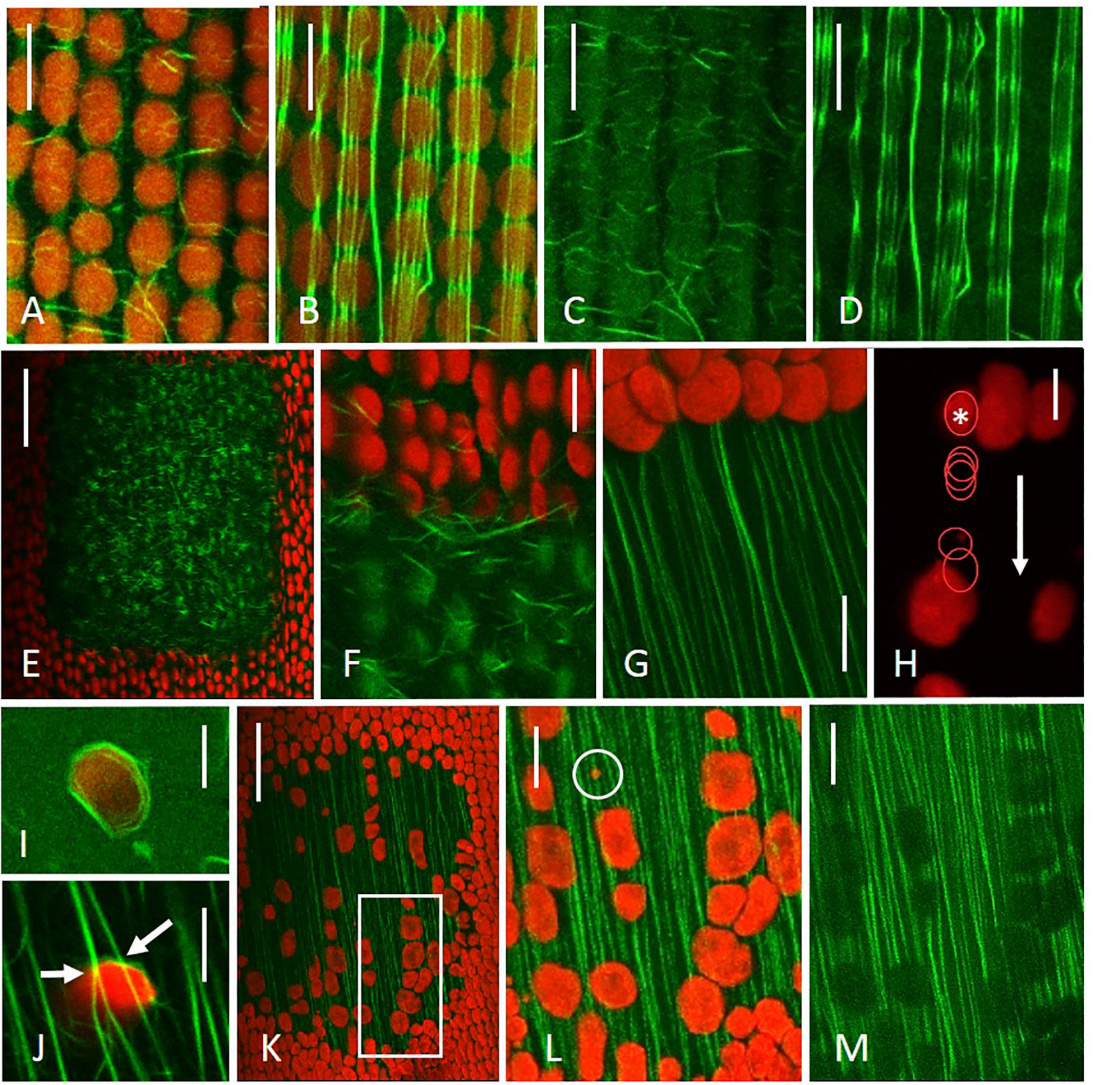
Actin cytoskeleton and chloroplasts in control regions and in windows. **(A–D)** Cortical actin strands **(A, C)** and subcortical actin bundles **(B, D)** in a control region. Note that the signal of the subcortical bundles is attenuated beneath chloroplasts **(D)**. **(E, F)** Short, randomly oriented actin rods are present between the plasma membrane and bleached chloroplasts. **(G)** After the detachment of bleached chloroplasts, subcortical actin bundles become clearly visible at the upstream end of the window. **(H)** At the cortex of a window, a chloroplast (asterisk and subsequent outlined stages) moves slowly and with irregular velocity parallel to the streaming direction (arrow). Time interval between stages is 5 s. The other chloroplasts were immobile during the observation period. **(I)** Actin ring around an endoplasmic chloroplast. **(J)** Contacts (arrows) between chloroplast-associated actin ring and subcortical actin bundles. Note that the signal of the subcortical bundles is not attenuated indicating that the chloroplast is still located in the endoplasm. **(K–M)** Actin filament bundles at the inner, endoplasmic side of resettled chloroplasts; chloroplasts are aligned parallel to the actin bundles. Inset in K corresponds to higher magnifications shown in **(L, M)**. A proplastid is seen in the encircled area **(L)**. Images were taken 2 hours **(E-F)**, 1 day **(H, J)**, 2 days **(G)** and 7 days **(K–M)** after irradiation. Images **(A–D, I)** were taken from control cells. Actin was visualized by perfusion of cells with green fluorescent phalloidin, red = chlorophyll fluorescence; **(A,B, E-G, I–L)** are merged images. Images are single optical sections, except **(G, J, K-M)** which are maximum projections of 27, 20, 100 and 75 optical sections with a thickness of 0.9 µm, respectively. Bars are 50 **(E, K)** and 10 µm (all other images).

### 3.3 Actin cytoskeleton and resettlement of chloroplasts

**Figures 4A–D** show the actin cytoskeleton in a control cell, stained by perfusion with fluorescent phalloidin. Delicate actin strands are present at the plasma membrane (**Figures 4A, C**); subcortical, continuous actin bundles extend along the inner, endoplasmic side of chloroplasts (**Figures 4B, D**). The fluorescence of these bundles is attenuated by the autofluorescence of the chloroplasts (**Figure 4D**).

Two hours after irradiation, numerous short actin strands and rods were present between the plasma membrane and bleached chloroplasts; they were distinct (mostly thicker and straighter) from the delicate actin strands in nearby control regions (**Figures 4E, F**). Subcortical actin filament bundles either persisted or regenerated within a few hours from the upstream (endoplasm-delivering) border of the window, bleached chloroplasts detached and cortical actin strands or rods disappeared (**Figure 4G**). After one day active endoplasmic streaming recovered to near-control rates over the whole window (**Supplementary Video 5**).

Most endoplasmic chloroplasts were passively and continuously transported within the flowing endoplasm with many of them actively rotating as previously described (**Supplementary Video 2**). At the cortex of windows, slow and discontinuous straight movement was occasionally observed (**Figure 4H**), which was never found in control regions. **Figures 4I, J** show actin filament bundles (rings) attached to the surface of endoplasmic chloroplasts. The actin ring in **Figure 4J** contacts two subcortical actin bundles in a window (arrows). A contact between an endoplasmic chloroplast without actin ring is shown in **Supplementary Figures 2C, D**. Such an orientation (plasma membrane – subcortical actin bundle – chloroplast) was, however, observed only rarely.

**Figures 4K–M** show resettled chloroplasts in a 7-days old window. Their bright chlorophyll fluorescence indicates that they are not remnants of the original chloroplast layer, but newly attached organelles. Chloroplasts were squeezed between plasma membrane and actin bundles just as in control regions (plasma membrane – chloroplast - subcortical actin bundle; **Figures 4A–D**), and chloroplast-associated actin rings were absent. Most subcortical bundles appeared to be continuous. However, since their fluorescence signal was attenuated by the chlorophyll fluorescence (**Figure 4M**), we cannot exclude the possibility that some bundles were interrupted. Subcortical bundles between resettled chloroplasts were close to the plasma membrane (see **Supplementary Figures 2E-F**). In regions with abundant chloroplasts and in fully replenished windows, fine actin strands were again present near the plasma membrane (**Supplementary Figure 2G**).

**Figure 5:**
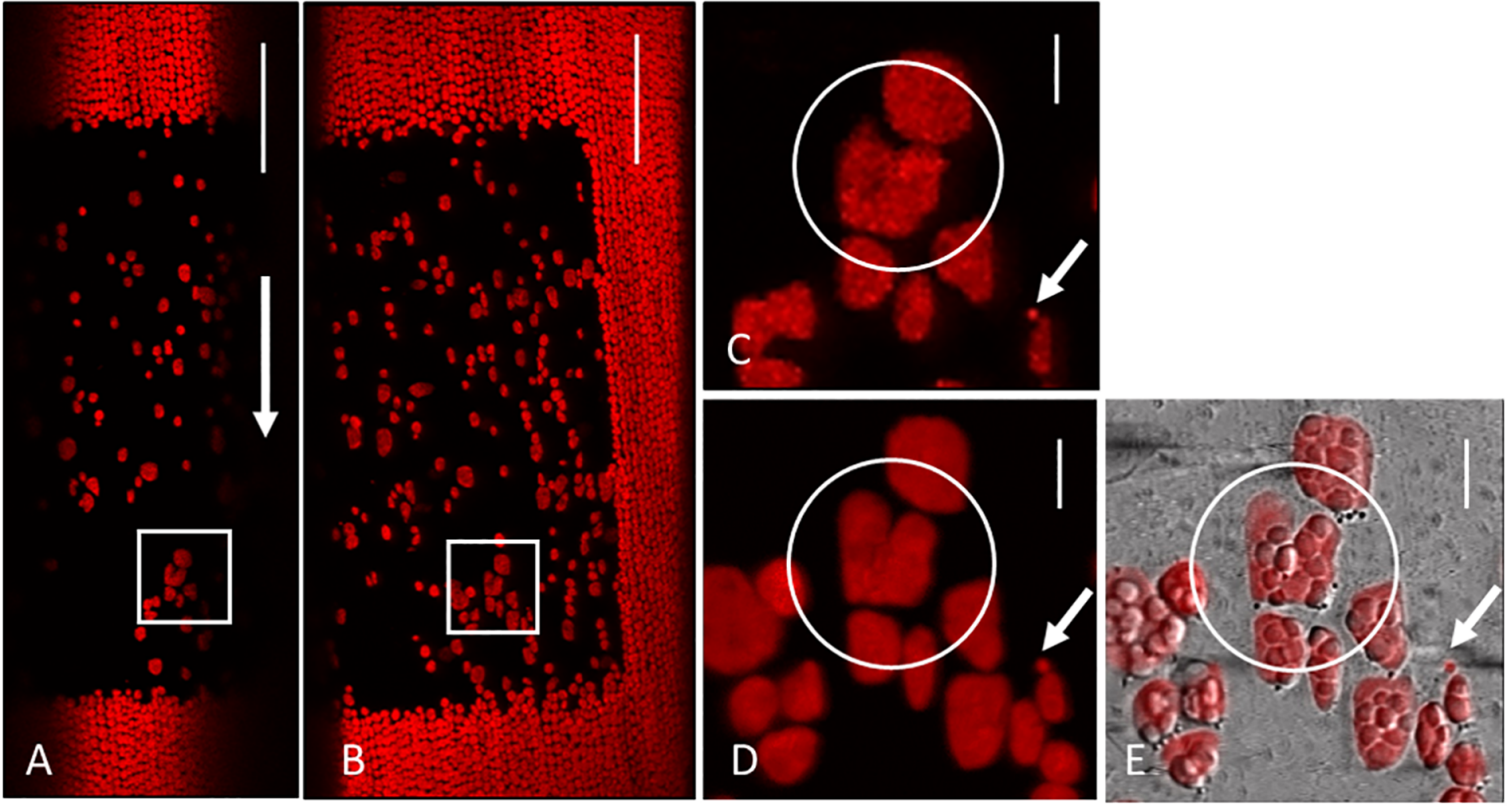
Giant chloroplasts resettled in a 5 **(A, C)** and 10 day old window **(B, D, E)**. The arrow in **(A)** indicates the streaming direction. The rectangles in **(A, B)** correspond to the enlarged images **(C–E)**. A chloroplast with a stable division furrow is encircled in **(C–E)**. The arrows in **(C–E)** mark the position of a proplastid. Chlorophyll autofluorescence **(A–D)**, merged with bright field **(E)**. Images **(A, B)** are maximum projections of 21 optical sections with a thickness of 0.9 µm, **(C–E)** are single sections. Bar = 50 µm **(A, B)** and 10 µm **(C, D, E)**.

Chloroplasts with division furrows were likewise able to resettle at windows and their shape remained unchanged for days (**Figures 5A–E**). **Figures 5C–E** (arrows) show that the size of a presumed proplastid did not increase during the observation period. Bleached chloroplasts were never observed to resettle at windows.

### 3.4 Preferred attachment of chloroplasts at the downstream end of windows

**Figure 6:**
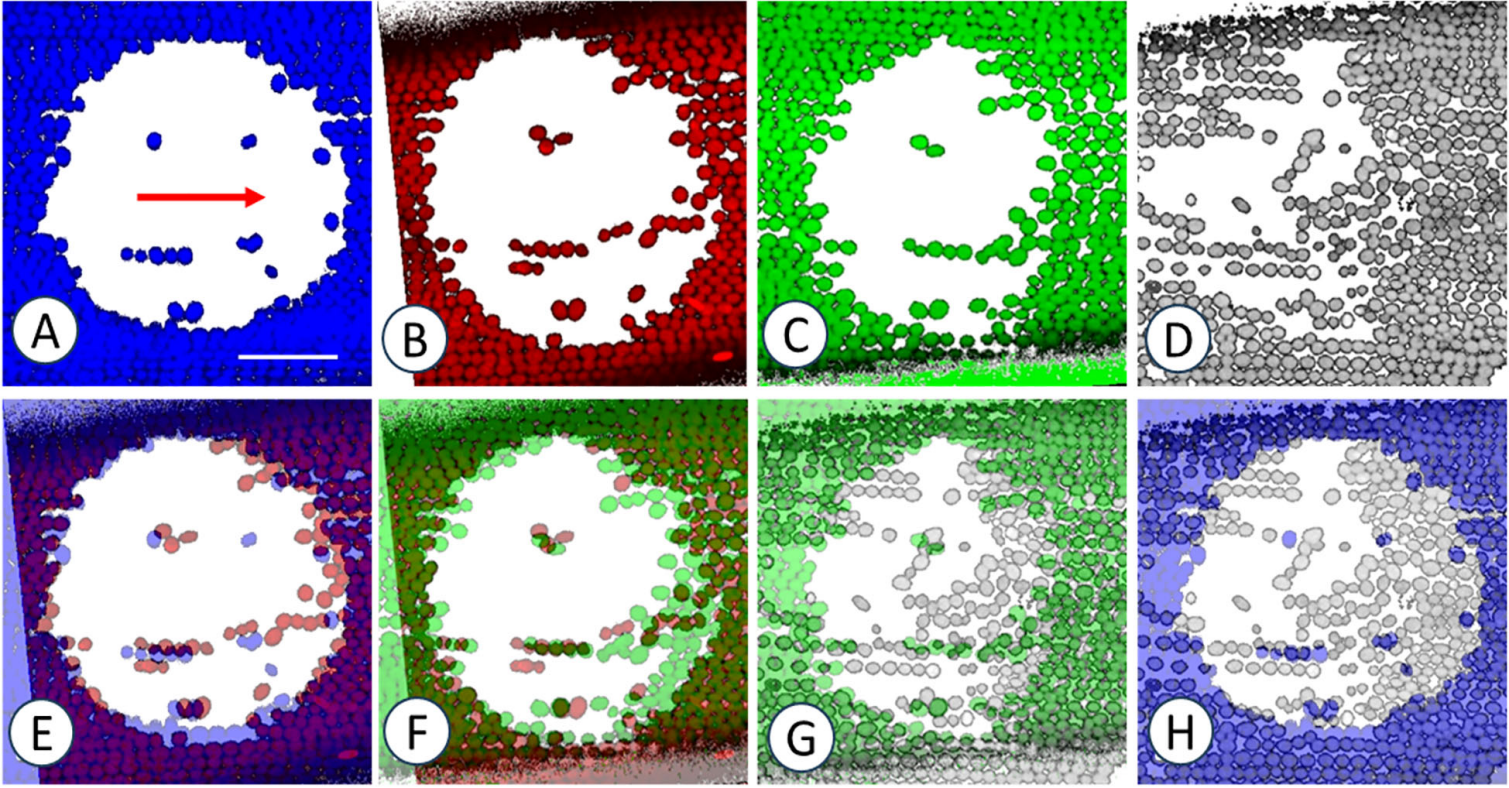
Preferred resettlement of chloroplasts at the downstream end of windows. Images were taken 1 day **(A)**, 1 week **(B)**, 3 weeks **(C)** and 5 weeks **(D)** after irradiation. Arrow in **(A)** indicates streaming direction. **(E–G)** Pairwise overlays of consecutive images (A/B, B/C, C/D). **(H)** Comparison between **(A, D)** False coloured images are maximum projections of 7 optical sections with a thickness of 1.3 µm. Bar in **(A)** = 50 µm for all images.

**Figure 7:**
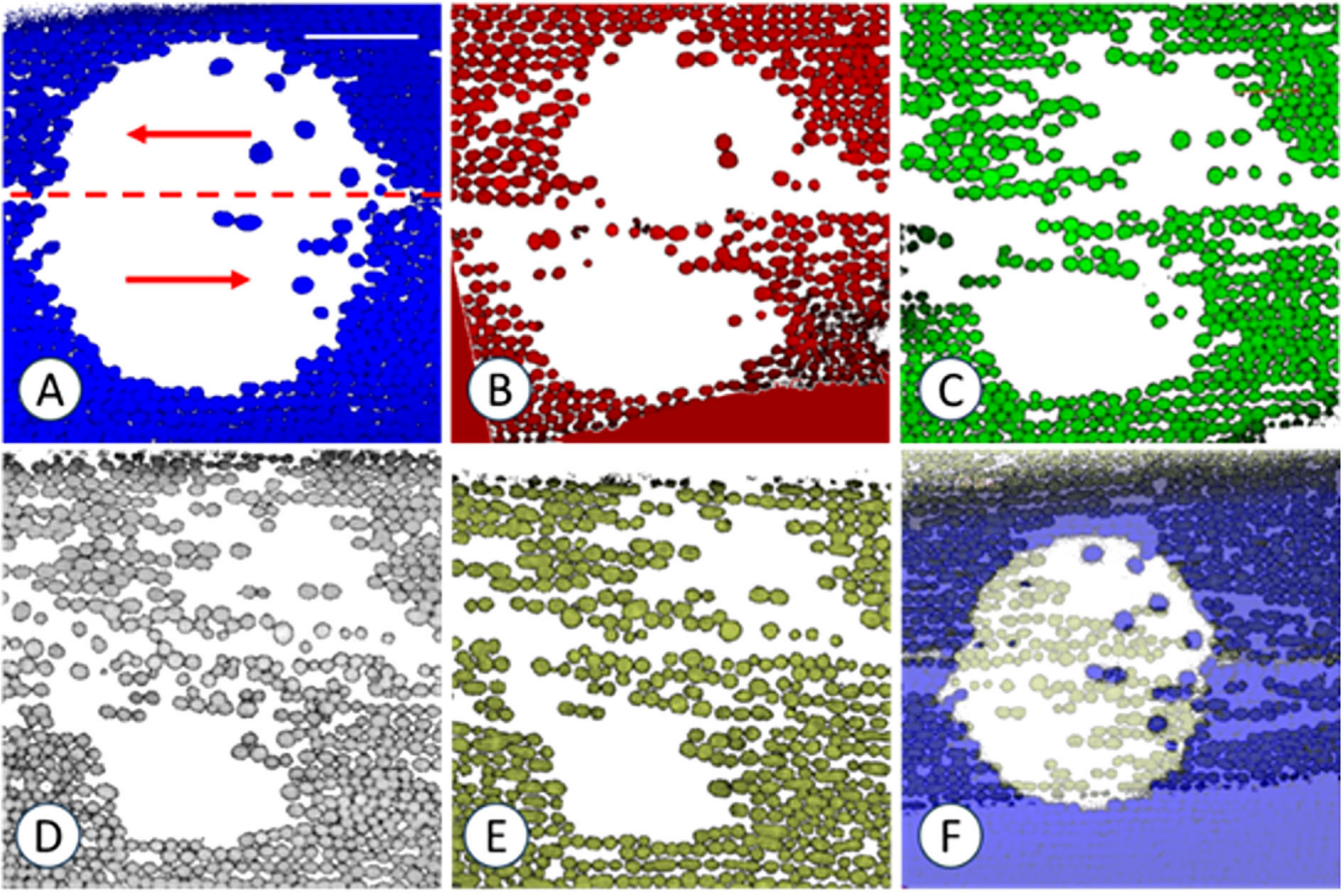
Resettlement of chloroplasts in a window located at the neutral line and in the presence of oryzalin. Images were taken 1 day **(A)**, 1 week **(B)**, 3 weeks **(C)**, 6 weeks **(D)** and 7 weeks **(E)** after irradiation. The cell was treated with 8 µm oryzalin before and after irradiation. Red arrows in **(C)** indicate streaming direction. **(F)** shows the overlay of **(A, E)** Note preferred resettlement of chloroplasts at the neutral line (dashed line in **A**) and at the two opposing downstream ends. False coloured images are maximum projections of 8 optical sections with a thickness of 1.3 µm. Bar in **(A)** = 50 µm for all images.

A typical time series illustrating the changes in chloroplast number and position over a period of 5 weeks is shown in **Figure 6**. The first image was taken one day after window formation with several scattered chloroplasts visible in the window. During the following days and weeks, some of them slightly changed their position, whereas others disappeared completely (**Figures 6A–D** and pairwise overlays in E-G). Chloroplasts often reattached in files parallel to the direction of actin bundles and cytoplasmic streaming, respectively (red arrow in A). The comparison of the first (A) and the last image of this series (D; see overlay H) reveals that endoplasmic chloroplasts resettled preferentially at the downstream end of the window. The preferred downstream attachment was clearly visible in windows containing the neutral line (**Figure 7**; note that this cell was also treated with microtubule depolymerizing oryzalin). Such windows show two opposing up- and downstream regions, respectively (arrows in **Figure 7A** indicate the streaming directions), with chloroplasts preferentially resettling at the corresponding downstream ends (compare **Figure 7F** which is the overlay of A and E). **Figure 7** also shows that not only the downstream ends are preferred sites for resettlement of chloroplasts, but also the neutral line itself. Observations in windows containing the neutral line also confirm the alignment of resettled chloroplasts along actin bundles. Inside windows or in their direct vicinity, the passive endoplasmic flow during irradiation became occasionally deviated across the neutral line which induces a U-shaped regeneration of the actin bundles and corresponding active endoplasmic flow. These U-turns are mirrored by the orientation of chloroplast files (**Supplementary Figure 3**).

**Figure 8:**
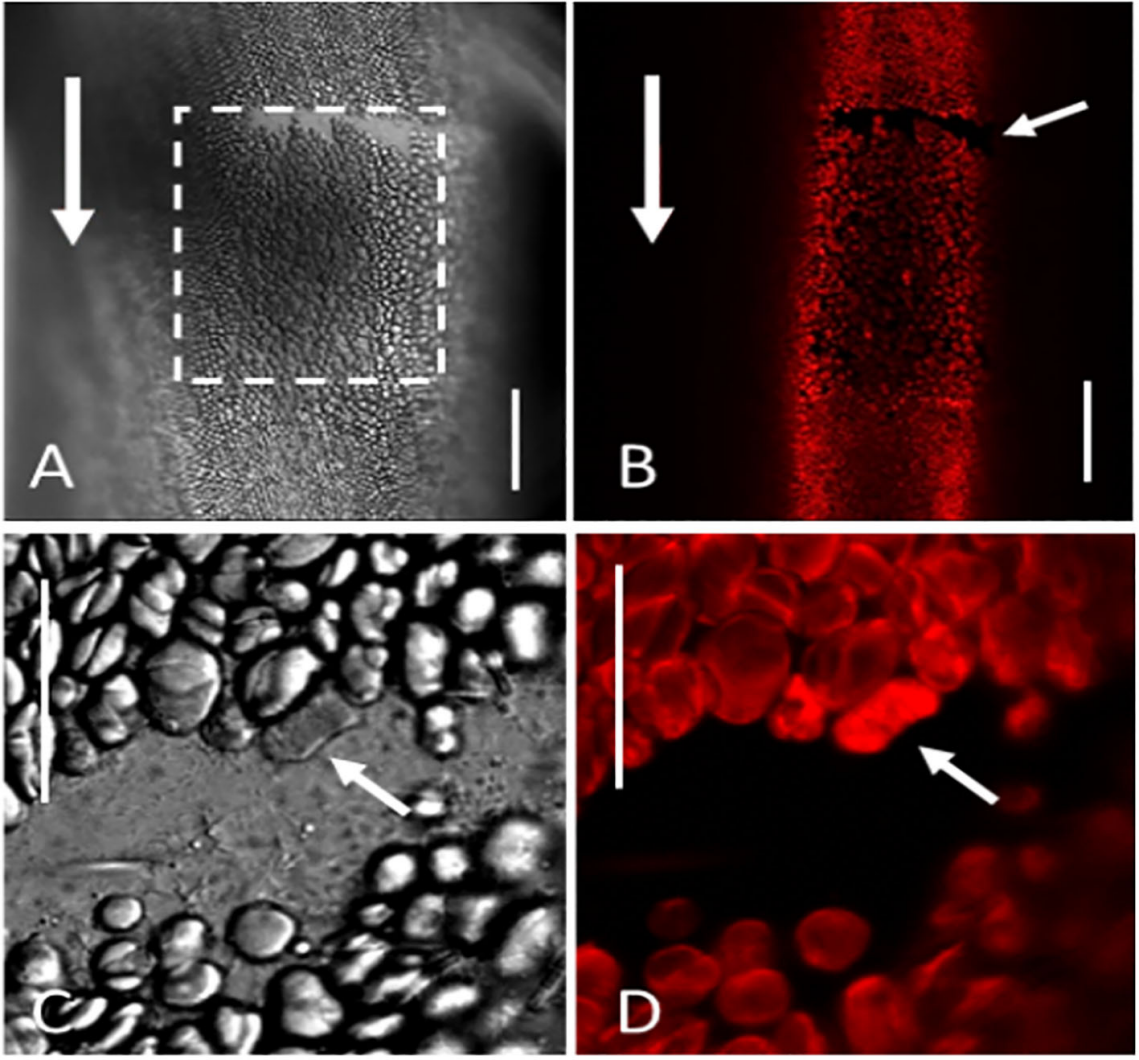
Healed (closed) window. The cell was irradiated 8 weeks before the image was taken. **(A, B)** Most of the irradiated area (rectangle in **A**) has been replenished by chloroplasts apart from a thin region at the upstream end of the window (small white arrow in **(B)**; large arrows indicate streaming direction). **(C, D)** Note starchless and stronger fluorescent chloroplast at the border of the window. **(A, C)** bright field, **(B, D)** chlorophyll fluorescence. All images are single sections. Bar = 100 µm **(A, B)** and 20 µm **(C, D)**.

Provided that the number of endoplasmic chloroplasts is sufficient, the entire window area will be covered by chloroplasts. These “closed” windows have parallel rows of chloroplasts and are almost indistinguishable from non-irradiated control areas (**Figures 8A, B**). Often, a narrow, sickle-shaped region at the upstream region remains chloroplast-free (small arrow in **Figure 8B**). The resettled chloroplasts accumulate starch grains, just as the control chloroplasts. We occasionally observed starch-free chloroplasts at the border of the window with more intense chlorophyll fluorescence signals compared to other chloroplasts (**Figures 8C, D**). We assume that these chloroplasts correspond to partly irradiated chloroplasts which did not detach (compare **Figure 2D**) and were partially damaged, which is in accordance with PAM fluorescence data (see below).

### 3.5 Actin-dependent cytoplasmic streaming, but not microtubules are required for release and resettlement of chloroplasts

**Figure 9:**
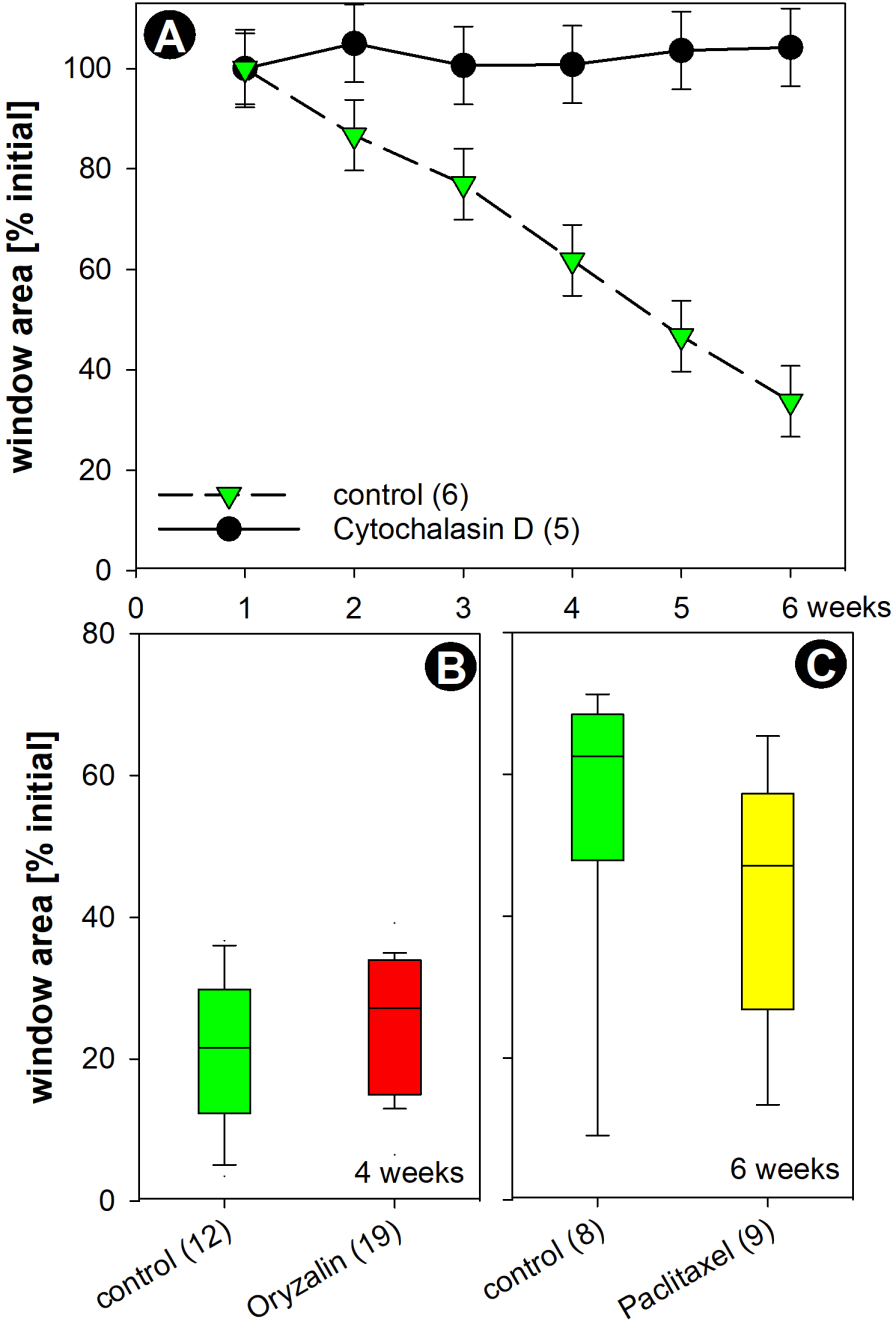
Effect of cytoskeleton inhibitors on the resettlement of chloroplasts. **(A-C)** Relative size of windows (chloroplast-free areas) in control cells (green symbols and green bars) and in cells treated with cytoskeleton inhibitors [black symbols in A, red and yellow bars in **(B, C)**]. Cells were irradiated and left in AFW for 1 day to allow the release of bleached chloroplasts. They were then treated with 20 µM cytochalasin D **(A)**, 8 µM oryzalin **(B)** or 10 µM paclitaxel **(C)**. Data are means ± SD, given as % of initial window size for **(A)** with GLMM resulting in significant differences (p< 0.001). For oryzalin (p = 0.54) and paclitaxel (p = 0.11), no significant differences were found (Mann-Whitney-test). Bars in **(B, C)** represent median boxes with 90-75-50-25-5 percentiles. Numbers in brackets indicate number of replicates.

To study the effect of cytoplasmic streaming we applied cytochalasin D. Concentrations of 10 and 20 µM cytochalasin D inhibited cytoplasmic streaming within 30 min (**Supplementary Video 6**) and, when applied before or immediately after bleaching, prevented the release of damaged chloroplasts (**Supplementary Figures 4A–C**; note inhibition of cytoplasmic streaming in **Supplementary Video 7** which is a z-stack of bright field images). The effect of cytochalasin D was reversible; chloroplast-free windows were obtained several hours after cytochalasin was washed out with AFW (**Supplementary Figures 4D–F**; note recovery of cytoplasmic streaming in **Supplementary Video 8** which is a z-stack of bright field images). When cytochalasin D was applied one day after irradiation, i. e. after the detachment of bleached chloroplasts, the resettlement of chloroplasts at windows was significantly impaired compared to the control (GLMM - groups p = 0.003, time p < 0.001, groups x time p < 0001; **Figure 9A**; **Supplementary Figures 4G–L**, **Supplementary Data Sheet 1**).

Depolymerization of microtubules by oryzalin (8 µM; **Figures 7A–F**, **9B**) or their stabilization by paclitaxel (10 µM; **Figure 9C**) had no significant effect on the recovery of the chloroplast layer (Mann-Whitney-U test p = 0.535 for oryzalin, p = 0.114 for paclitaxel, **Supplementary Data Sheet 1**).

### 3.6 Photosynthetic performance of chloroplasts

**Figure 10:**
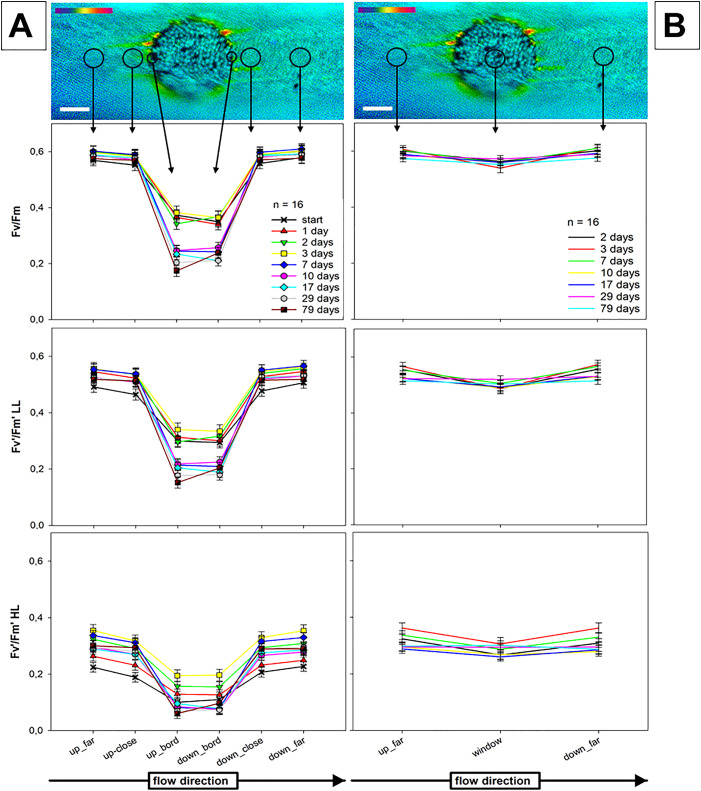
Photosynthetic parameters of chloroplasts in branchlet internodal cells of *C. australis*. Fluorescence yields measured after dark adaptation (Fv/Fm) and after exposure to low light (20 µmol photons m^-2^.s^-1^; Fv’/Fm’ LL) and high light (60 µmol photons m^-2^.s^-1^; Fv’/FM’ HL), respectively. The top image shows a false colour image of maximum dark yield (Fv/Fm) of resettled chloroplasts, damaged chloroplasts at the border of the window and chloroplasts in undamaged control regions. Arrows indicate measuring spots for the graphs placed below. **(A)** Comparison between chloroplasts located at the border of the window and chloroplasts in control regions. **(B)** Comparison of chloroplasts resettled at windows and chloroplasts in remote regions. Means and standard errors. Bar in **(A)** = 50 µm.

We determined photosynthetic performance in resettled and control chloroplasts using PAM fluorescence. Fluorescence yields were measured over a period of up to 78 days after irradiation took place. Measuring points included control regions about 100 µm (close) and 200 µm (far) apart from the window, resettled chloroplasts and individual chloroplasts directly at the border of the windows (“border chloroplasts”) (**Figure 10**).

**Figures 10A, B** show Fv/Fm, Fv’/Fm’ LL and Fv’/FM’ HL. **Figure 10A** displays the comparison between control chloroplasts outside the window and border chloroplasts. Both Fv/Fm and Fv’/Fm’ of border chloroplasts were always significantly lower than those of control chloroplasts and further decreased with time (GLMM, **Supplementary Data Sheet 2**). No significant differences were found between control regions located close or far away from the window or located at the upstream or downstream end of windows. **Figure 10B** compares the properties of resettled chloroplasts and chloroplasts in control regions. The data obtained for resettled chloroplasts were slightly, but significantly lower than those measured in control chloroplast, independent of light provided (GLMM, **Supplementary Data Sheet 3**). Fv/Fm and Fv’/Fm’ values of resettled chloroplasts remained stable over time, but values of control chloroplasts slightly decreased.

### 3.7 Detection of *CaCHUP1* and analyses of its protein sequence

CHUP1 is involved in chloroplast movement in vascular plants (see Kong et al., 2024 and references therein). We screened for this protein in *Chara* databases and found homologous forms in *C. australis* as well as in *C. braunii*. To examine the sequence more closely, we cloned and sequenced the CHUP homolog of *C. australis*. CaCHUP1 comprises 1201 amino acids and has a calculated MW of 133 kDa. Both *Characean* sequences were aligned with representative proteins of *Klebsormidium nitens* (KnCHUP1B, Charophyta), *Marchantia paleacea* (MpCHUP, liverworth), *Physcomitrium patens* (PpCHUP, moss), *Adiantum capillus-veneris* (AcvCHUP1A, fern), and the eudicot *Arabidopsis thaliana* (AtCHUP1); conserved domains and regions were labelled (see **Supplementary Figure 5**). While the two proteins of *Chara* (CaCHUP1 and the hypothetical protein CBR_g4598 of *C. braunii* (named CbCHUP in the alignment) showed 92% sequence homology when compared to each other, CaCHUP1 shared only 26-29% identity when compared to all other CHUP sequences mentioned above.

An InterPro search revealed domains and regions all sharing an N-terminal signal peptide sequence that directs them to the outer membrane of chloroplasts (**Supplementary Figure 5** illustrates their arrangement along the proteins. They all share an N-terminal signal peptide sequence that directs them to the outer membrane of chloroplasts. Moreover, all sequences show a coiled-coil region followed by an F-actin-binding region at the beginning of CHUP1/IPGA1-like domain (IPR040265, CHLOROPLAST UNUSUAL POSITIONING 1/INCREASED PETAL GROWTH ANISOTROPY 1). Interestingly, all three *Charophyta* protein sequences show slightly longer coiled-coil and CHUP-domains.

**Figure 11:**
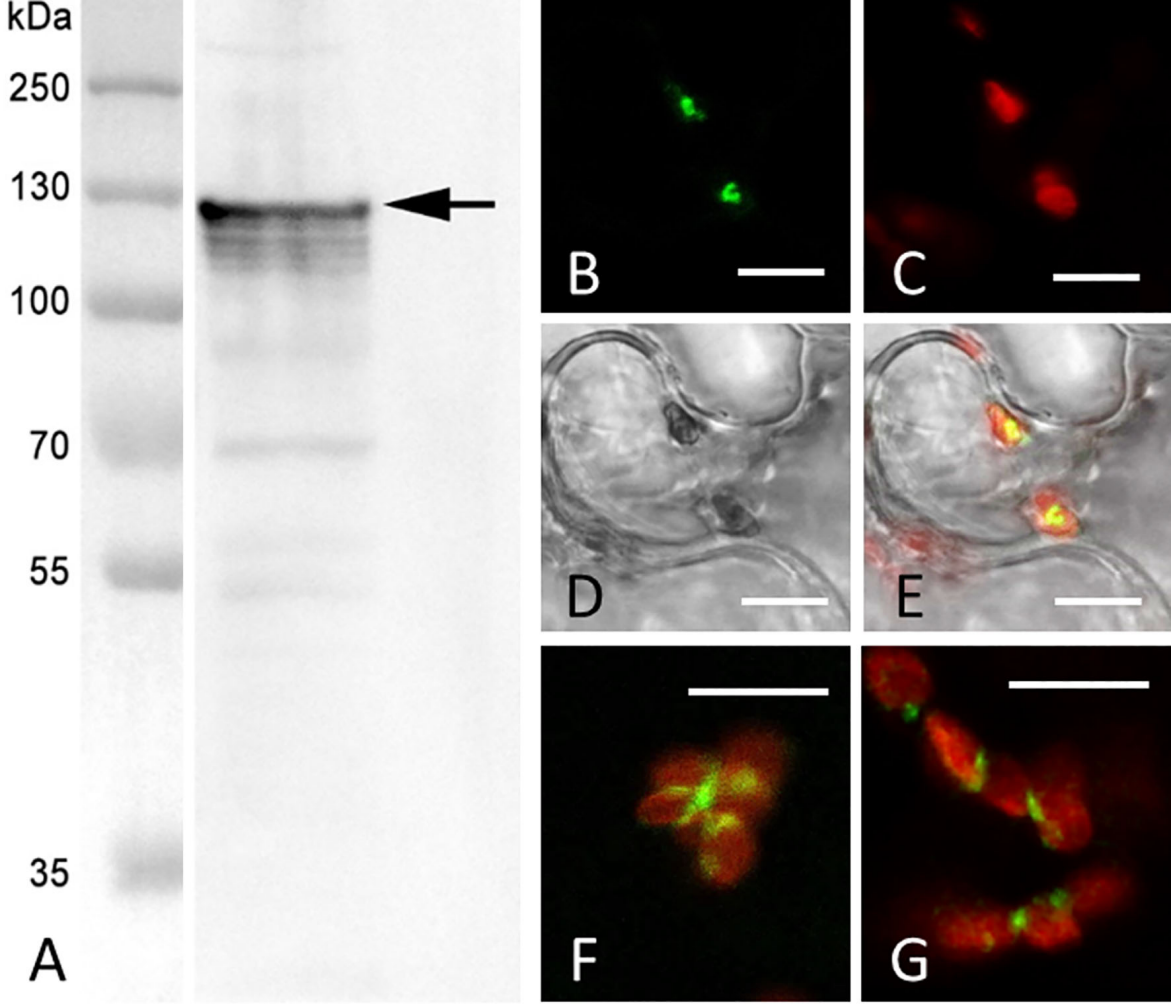
Visualization of native CaCHUP1 in *C*. *australis* protein extract and localization of transiently expressed CaCHUP1-GFP in tobacco leaves. **(A)** Western blot of *C*. *australis* protein extract. The prominent CaCHUP1 band (arrow) was visualized using an antibody created against the protein sequence of CaCHUP1 (marker = molecular mass in kDa) **(B–G)** Transient expression of CaCHUP1-GFP in epidermal cells of tobacco leaves. **(B, C)** CaCHUP1-GFP is shown in green, while chloroplasts are shown in red (chlorophyll autofluorescence); **(D)** is the corresponding DIC image which is merged with the fluorescent images in **(E)** In **(F, G)** the GFP fluorescence is merged with chlorophyll fluorescence. All images are single sections. Bars are 10 µm.

Native CaCHUP1 protein was visualized by western blot analyses using *C. australis* protein extracts and an antibody created against the protein sequence of CaCHUP1 (see **Figure 11A**). A prominent band was visible at about 130 kDa, as expected from the calculated molecular mass of the protein.

At present, we are not able to transform *Chara* directly. Thus, we expressed CaCHUP1 in tobacco leaves to gain insights into its potential function. As shown in **Figures 11B–G**, CLSM revealed a CaCHUP1-GFP that localizes in the form of small, punctate spots on the surface of chloroplasts.

## 4 Discussion

### 4.1 Chloroplasts of *Chara* internodal cells

In contrast to most vascular plants and non-motile algae (Suetsugu et al., 2017; Wada, 2013), the chloroplasts of characean internodal cells are firmly anchored at the cell periphery. They are embedded in a fibrous meshwork (presumably cortical actin and associated filaments) between the plasma membrane and the subcortical actin bundles which run parallel to the chloroplast files (Wasteneys et al., 1996; Williamson, 1985; Williamson et al., 1985). Dissolution of the presumed actin meshwork by perfusion of cells with low-ionic strength (low salt) solution leads to a loss of the subcortical actin bundles and releases the chloroplasts. These observations suggest that the close association of actin with chloroplasts reflects a dependence of chloroplast alignment on actin bundle organization (e.g. Wasteneys et al., 1996). *Chara* chloroplasts must therefore be equipped with proteins responsible for anchorage at the plasma membrane, with actin-binding and, possibly, actin-polymerizing proteins. In addition, the cortical ER was also shown to participate in the immobilization and relocation of chloroplasts (Andersson et al., 2007a, b; Lichtscheidl and Url, 1990).

Chloroplasts may detach from the cortex and are then transported along with endoplasmic flow; reasons for their release are manifold. From time to time, long-living giant chloroplasts in the endoplasm are present, which probably reflects an imbalance between cell elongation and chloroplast growth and division (compare Green, 1964). Chloroplasts may be released after mechanical damage, centrifugation, chemical treatment (references in Foissner and Wasteneys, 2012; 2014), and local illumination with excess light (“window” formation; Kamitsubo, 1972). In the current study, strong blue light was provided for window formation, but also UV (Foissner and Wasteneys, 2012), green, yellow and red light (laser lines 514, 561 and 633, respectively) of adequate duration and intensity can be used (unpublished observations), which indicates that detachment is not linked to a photoreceptor (Łabuz et al., 2022). It still remains an enigma, how irradiation causes a loosening of chloroplast anchoring. One possible explanation suggests a role of Ca^2+^ binding proteins, because Foissner and Wasteneys (2012) reported that chemicals causing an influx of Ca^2+^ into the cell induce the release of chloroplasts.

**Figure 12:**
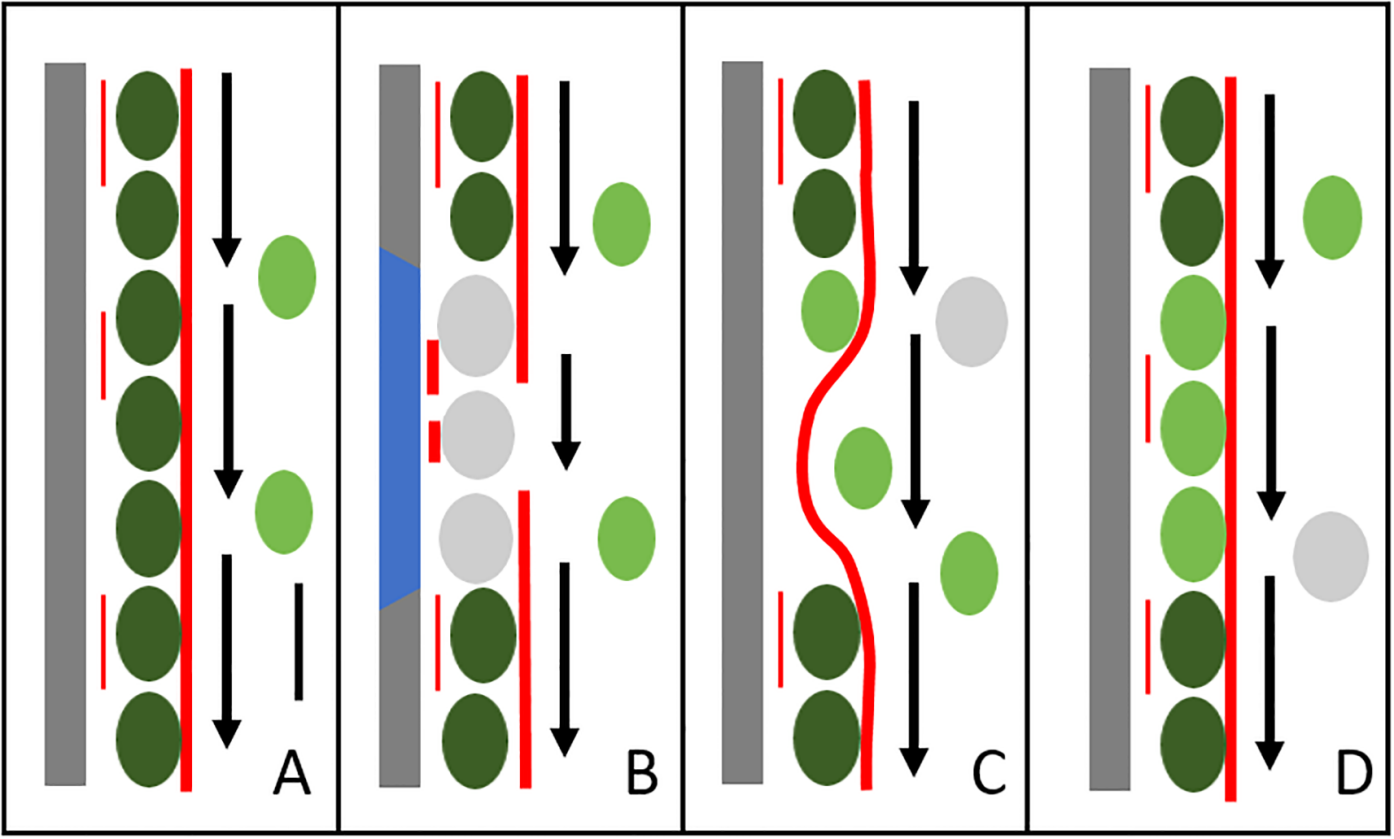
Schematic illustration of actin-dependent chloroplast resettlement at windows created in *Chara* internodal cells **(A)** Intact cell. Stationary, cortical chloroplasts (dark green) are located next to the cell wall (grey bar), thin red lines are cortical actin filaments near the plasma membrane, thick red line is a subcortical actin bundle. Endoplasmic chloroplasts (light green) are passively transported in the endoplasm (arrows indicate direction and velocity). **(B)** Irradiation with strong light (blue bar) causes bleaching and swelling of cortical chloroplasts (grey) and the transient formation of short cortical actin bundles. Endoplasmic streaming is locally disturbed and, possibly, subcortical bundles are disrupted. **(C)** Recovery of subcortical actin bundles and endoplasmic streaming after detachment of bleached chloroplasts. Note that subcortical bundles are closer to the plasma membrane. **(D)** Replenished window. Only organelles and structures relevant for this process are drawn. Bar in **(A)** = approximately 10 µm.

We were able to show that non-damaged endoplasmic chloroplasts resettle in windows, leading to an almost complete structural and physiological repair of the cortical layer (see **Figure 12** for a schematic summary). Long-term observations of individual resettled chloroplasts, including presumed proplastids (**Figure 5**; Choi et al., 2021), and the fact that the repair of the cortical chloroplast layer depends on the presence of endoplasmic chloroplasts (**Figures 2A–C**) exclude the possibility that chloroplast division is involved. Bleached chloroplasts released from irradiated areas were not able to recover and to re-attach at windows.

### 4.2 Actin cytoskeleton is involved in the release and resettlement of chloroplasts

The actin cytoskeleton, especially cytoplasmic streaming, plays a pivotal role both in the removal of bleached chloroplasts and in the resettlement of intact chloroplasts. After treatment with inhibitory concentrations of cytochalasin D, bleached chloroplasts remained in place and, if applied after removal of bleached chloroplasts, windows remained empty (**Supplementary Figures 4A–C**; **Figure 9A**). Both effects were reversible (**Supplementary Figures 4D-F**). Depolymerization of microtubules by oryzalin or stabilization by paclitaxel had no significant effect on the recovery of the cortical chloroplast layer (**Figures 9B, C**). Previous studies have shown that microtubules are also not required for the deposition of a wound wall after mechanical or chemical damage (Foissner and Wasteneys, 2012).

The recovery of the actin cytoskeleton in windows was already described earlier (e. g. Williamson et al., 1984) and is summarized in **Figure 12** which includes the findings obtained in this study. Actin filament bundles regenerate from the upstream region of the window which corresponds to the growing (barbed) end of actin filaments (Williamson et al., 1984). In contrast, chloroplast resettlement preferably occurs at the downstream border of windows. This is possibly due to the delayed recovery of actin bundles which retards cytoplasmic bulk streaming (just as at the neutral line; **Figure 7**) and thereby facilitates the physical contact of the chloroplasts with the cortex. Furthermore, chloroplasts arriving at the downstream end encounter an “obstacle” in form of the cortical chloroplasts at the edge of the window. This will also lead to a disturbance and retardation of laminar streaming, accumulation of chloroplasts and probably closer contact with the cortex.

Once a chloroplast is settled, the probability that other chloroplasts attach near or at the upstream end of this chloroplast is high. This leads to the formation of chloroplast files parallel to the direction of mass streaming or subcortical actin bundles, respectively. In principle, chloroplasts may attach not only at the downstream end but anywhere in the window and serve as a starting point for the formation of a file by consecutive alignment of individual plastids.

In the absence of resettled chloroplasts, the cortex at windows is shielded from the endoplasm by a dense layer of subcortical actin bundles which are located close to the plasma membrane (**Figure 4G**; **Supplementary Figures 2E-G**); compare Foissner and Wasteneys, 1999). Saltatory, probably active movements of chloroplasts at the window as well as contact between chloroplast-associated actin rings with subcortical bundles have been observed (**Figures 4H, I**). We assume that they are due to actin-actin interactions either via bundling proteins (e.g. formin; Thomas et al., 2009), motor proteins (myosin; Buchnik et al., 2015; Ueda et al., 2015), or proteins involved in actin polymerization (e. g. CHUP1; see below). The chloroplasts have then to cross the actin layer to get their final position near the plasma membrane. Whether this involves a slipping between actin bundles or, more probably, dynamic actin remodeling (de- and repolymerization of actin bundles) remains to be investigated.

Chen (1983) mentioned that chloroplasts align along (abnormal) pathways of streaming after mechanical removal of cortical chloroplasts by means of centrifugation. We identified the same phenomenon by providing excess light. In addition, we studied chloroplast resettlement in detail, which has not been published before.

### 4.3 Photosynthetic performance of chloroplasts

Chlorophyll fluorescence measurements showed that the photosynthetic properties of resettled chloroplasts are within the range of those in non-damaged regions. Thus, the cortex at windows recovered not only in the typical morphology, also the photosynthetic performance was comparable to the control regions. Chloroplasts with extremely low Fm/Fv and Fm’/Fv’ values, which decreased further with time, were occasionally found at the border of the windows (**Figure 11**). They probably corresponded to partly bleached chloroplasts which were neither able to detach nor able to recover, consistent with the absence of starch grains (**Figures 8C, D**). These impacted chloroplasts, however, survived the whole experiment for around three months.

Bulychev and coworkers (Bulychev, 2019, 2020; Dodonova and Bulychev, 2011) found that chloroplast performance measured at some distance of a local light spot (which did not cause chloroplast release), varied according to the direction of cytoplasmic streaming, with lower fluorescence values downstream the irradiated area. They concluded that ions and metabolites released during photosynthesis affect the performance of chloroplasts in shaded regions. Based on these studies, we also considered the possibility of a cyclosis-driven asymmetry along the illuminated measuring area: upstream endoplasm might have a different chemical composition than the downstream endoplasm just before leaving the illuminated area. In this study, however, we did not find any significant differences in fluorescent yields, neither between the reference spots, nor between up- and downstream window margins. Our results indirectly confirm the experiments of Bulychev (2019, 2020), because photosynthetic metabolites are not released in the absence of chloroplasts (window area). In addition, distances between chloroplast-bearing control spots might have been too small for measuring differences, but this assumption calls for further investigation.

### 4.4 Chloroplast anchorage and actin binding proteins

Characean chloroplasts have characteristics comparable to those of vascular plants, to which they are closely related (e.g. McCourt et al., 2011; Turmel et al., 2013). In vascular plants (Wada and Kong, 2018) and probably also in ferns, mosses, and certain algae (e.g. Mineyuki et al., 1995), a protein complex is involved in both motility and anchorage of chloroplasts. Among these proteins, CHUP1 is required for chloroplast photo-relocation. CHUP1 homologs were found in *Nitella mirabilis* (Suetsugu and Wada, 2016) and also in the current study in *C. australis*. Recently, Yamamoto-Negi et al. (2024) characterized a CHUP1 in *Marchantia polymorpha* and pointed out the relevance of this protein in chloroplast positioning. Very recently, CHUP1 was also shown to restrict movement of chloroplasts in tobacco and *Arabidopsis* (Nedo et al., 2024). CaCHUP1, like other known CHUP1 proteins, is composed of several functional domains and regions (compare **Supplementary Figure 5**) including the N-terminal hydrophobic signal peptide that targets the protein to the outer membrane of chloroplasts (compare **Figures 11B-G**). The signal peptide is followed by a region of several coiled-coil domains that is described to function in anchoring chloroplasts to the plasma membrane. On the C-terminus, the highly conserved CHUP1/IPGA1-like domain with its actin-binding region is located. Both, N-terminal hydrophobic signal peptide and CHUP1/IPGA1-like domain, are described to be involved in movement and proper positioning of chloroplasts in plants (Oikawa et al., 2003; 2008). The presence of orthologs of KAC in the Charophyceae (Suetsugu and Wada, 2016) suggests that CaCHUP1 is also involved in actin polymerization. A role of CaCHUP1 in chloroplast motility can be excluded since *Chara* chloroplasts are immobilized in the cortex under normal conditions. When released from the cortex, the organelles are passively transported by bulk endoplasmic flow. Actin rings or polygons at the surface of chloroplasts support active rotation (**Figures 4I, J**; Wasteneys et al., 1996), but clusters of short actin filaments – typical for cp-actin (Kong et al., 2024) – have never been observed in characean internodal cells (compare Wasteneys et al., 1996; Williamson and Hurley, 1986; Williamson et al., 1987). These observations, along with the fact that *Chara* chloroplasts cannot perform active photo-relocation, makes movement via polymerization of actin unlikely. It is more plausible that rotation and possible short-range movements of chloroplasts at windows occur via actin-myosin interaction, similar to endoplasmic streaming (Grolig et al., 1988). Even for vascular plants, active movement of plastids along actin bundles with the help of myosin motors is still a topic of discussion (e.g. Erickson and Schattat, 2018; Krzeszowiec and Gabryś, 2007; Natesan et al., 2009; Paves and Truve, 2007; Suetsugu et al., 2010) and furthermore, the exact mechanism of myosin-dependent motility in plants remains unclear (Buchnik et al., 2015).

Recent work indicates that chloroplasts in *Nicotiana* epidermal cells are transported via cytoplasmic streaming and that NtCHUP1 is involved in chloroplast anchorage (Nedo et al., 2024). We assume that CaCHUP1 plays a comparable role in the polymerization of actin filaments and bundles (but not of cp-actin) and in the anchorage of chloroplasts together with other proteins. Our study revealed that chloroplasts use different strategies for re-localization, either via polymerization of specialized cp-filaments or via cytoplasmic streaming. More information about the function of CaCHUP1 will be gained once a transformation method for characean internodes has been developed.

## Data Availability

The datasets presented in this study can be found in online repositories. The names of the repository/repositories and accession number(s) can be found in the article/**Supplementary Material**.

## Supplementary material

Supplementary Figure 1 Organelle motility in window and control region. **(A, B)** Bright field image and fluorescence image of a window (indicated by dashed line) two hours after irradiation. **(C)** Trajectories of endoplasmic (non-bleached) chloroplasts in the window and in the adjacent control region. Arrows indicate direction and distance travelled within 1.3 seconds. The short arrows within the window reflect lower velocities. Bars are 100 µm

Supplementary Figure 2 Detached chloroplasts, actin cytoskeleton and resettled chloroplasts. **(A, B)** Clump of bleached, detached chloroplasts (arrows) in the endoplasm of a cell 1 day after local irradiation. A is the bright field image and B the corresponding fluorescence image. **(C, D)** Actin cytoskeleton and resettled chloroplasts in a 4 day old window. The arrow points at an actin bundle located between an endoplasmic chloroplast and the cell periphery. The fluorescence signal of the other actin bundles is attenuated beneath chloroplasts (e. g. asterisk) indicating that they are located along the inner, endoplasmic side of chloroplasts. **(E)** Subcortical actin bundles at a 1-week old window. **(F)** Depth coding image of the area indicated by an arrow in the z stack E. Purple-coloured actin bundles are close to the plasma membrane, yellow-coloured actin bundles are located at the inner, endoplasm-facing side of a resettled chloroplast (arrow). **(G)** Actin filaments in the cortex of a replenished window (image was taken 4 weeks after irradiation). Actin was visualized by green fluorescent phalloidin **(C, H)** and merged with red chlorophyll fluorescence in D and E. Images are single sections **(A, B, G)** and maximum projections of 20 **(C, D)** and 12 **(E)** optical sections with a thickness of 0.9 µm **(C, D)**. Bars are 50 µm **(A, B)**, 20 µm **(E)**, 5 µm **(C, D, F, G)**.

Supplementary Figure 3 Orientation of resettled chloroplasts determined by passive endoplasmic flow. In windows located near the neutral line (NL) subcortical actin bundles and chloroplast files occasionally regenerate into the opposite streaming direction (lower arrow) along the passive cytoplasmic flow prevailing after irradiation. Image is a maximum projection of 15 optical sections with a thickness of 1.3 µm. Bar = 100 µm

Supplementary Figure 4 Inhibition of endoplasmic streaming by cytochalasin reversibly delays the detachment of bleached chloroplasts and prevents the resettlement of endoplasmic chloroplasts. **(A-E)** Cell was treated with 10 µM cytochalasin D during and after irradiation; images A-C were taken 1 week after irradiation. Images D and E show the same window 1 day after recovery in AFW. **(F)** Area covered by bleached chloroplasts in % of irradiated area (window) in cells treated as described above. Data are means±SD, Asterisk indicates significant differences (P≤0.05; t-test; 22 cells from 6 different thalli were investigated). **(G-L)** Cell was irradiated and remained in AFW to allow the detachment of irradiated chloroplasts. After 1 d the cell was incubated in 10 µM cytochalasin D over a period of 7 weeks (compare **Figure 9**). Images A and D are maximum projections of 5 and 7 optical sections with a thickness of 1.3 µm; images B,C and E are single sections. Images G-L are false coloured projections of chlorophyll fluorescence (22 optical sections with a thickness of 1.36 µm) and were taken 1 day **(G)**, 1 week **(H)**, 3 weeks **(I)**, 5 weeks **(J)** and 7 weeks **(K)** after irradiation. **(L)** is the comparison between the first **(D)** and the last picture **(H)** of this series. Bars are 50 µm.

Supplementary Figure 5 Protein sequence alignment of different plant CHUPs. The alignment was performed with Clustal Omega (EMBL-EBI). Identical residues are highlighted in black, and numbering of amino acid residues begins at the first methionine. The following sequences were compared: Charophytes: Chara australis CHUP1 (CaCHUP1, this paper), Chara braunii hypothetical protein CBR_g4598 (CbCHUP, GenBank: GBG69767), Klebsormidium nitens CHUP1B (KnCHUP1B, GenBank: GAQ78600); liverworth: Marchantia paleacea hypothetical protein Mapa_014704 (MpCHUP, GenBank: KAG6543864); moss: Physcomitrium patens CHUP1 isoform X1 (PpCHUP, NCBI Reference Sequence: XP_024379808); fern: Adiantum capillus-veneris CHUP1A (AcvCHUP1A, GenBank: BAG54845); eudicot: Arabidopsis thaliana CHUP1 (AtCHUP1, GenBank: OAP01771); conserved domains and regions are displayed in different colours as indicated.

Supplementary Data Sheet 1 Statistics results of inhibitor experiments with cytochalasin, oryzalin, paclitaxel.

Supplementary Data Sheet 2 Statistics results of PAM fluorescence measurements control regions and border chloroplasts.

Supplementary Data Sheet 3 Statistics results of PAM fluorescence measurements control regions and resettled chloroplasts.

Supplementary Video 1 Rotational streaming in an internodal cell of *Chara australis* (chlorophyll fluorescence and bright field images). Note neutral line with opposite flow directions of the endoplasm.

Supplementary Video 2 Giant rotating chloroplasts in the stagnant endoplasm at the neutral line (compare **Figures 2D-F**). Red chlorophyll fluorescence is merged with bright field image.

Supplementary Video 3 Slowly streaming endoplasm 1 hour after local irradiation (Red chlorophyll fluorescence, bright field and merged images; compare **Figures 2G-I**).

Supplementary Video 4 Wiggling motion of bleached chloroplasts 5 hours after irradiation.

Supplementary Video 5 Recovery of continuous endoplasmic streaming at a window 1 day after local irradiation.

Supplementary Video 6 Reversible inhibition of endoplasmic streaming by cytochalasin D (bright field images). Combined time series of an internodal cell taken before (left), 30 minutes after treatment with 20 cytochalasin D (middle) and after 1 hour recovery in artificial fresh water (right).

Supplementary Video 7 Cytochalasin D inhibits the detachment of bleached chloroplasts. Cell was treated with 10 µM cytochalasin D during and after irradiation, images were taken 1 week after bleaching. Note stagnant endoplasm in the Z-stack of bright field images corresponding to **Supplementary Figures 4A-C**.

Supplementary Video 8 Detachment of bleached chloroplasts after 1-day recovery from treatment with cytochalasin D in AFW (see **Supplementary Video 7**). Note recovery of endoplasmic streaming in the Z-stack of bright field images corresponding to **Supplementary Figures 4D, E**.
