# Supplementary figures and images for: Recovery of the cortical chloroplast layer in the green alga Chara after local irradiation
Source: Front Plant Sci. 2025 May 5;16:1544999. doi: 10.3389/fpls.2025.1544999 (PMC12086157; doi:10.3389/fpls.2025.1544999)

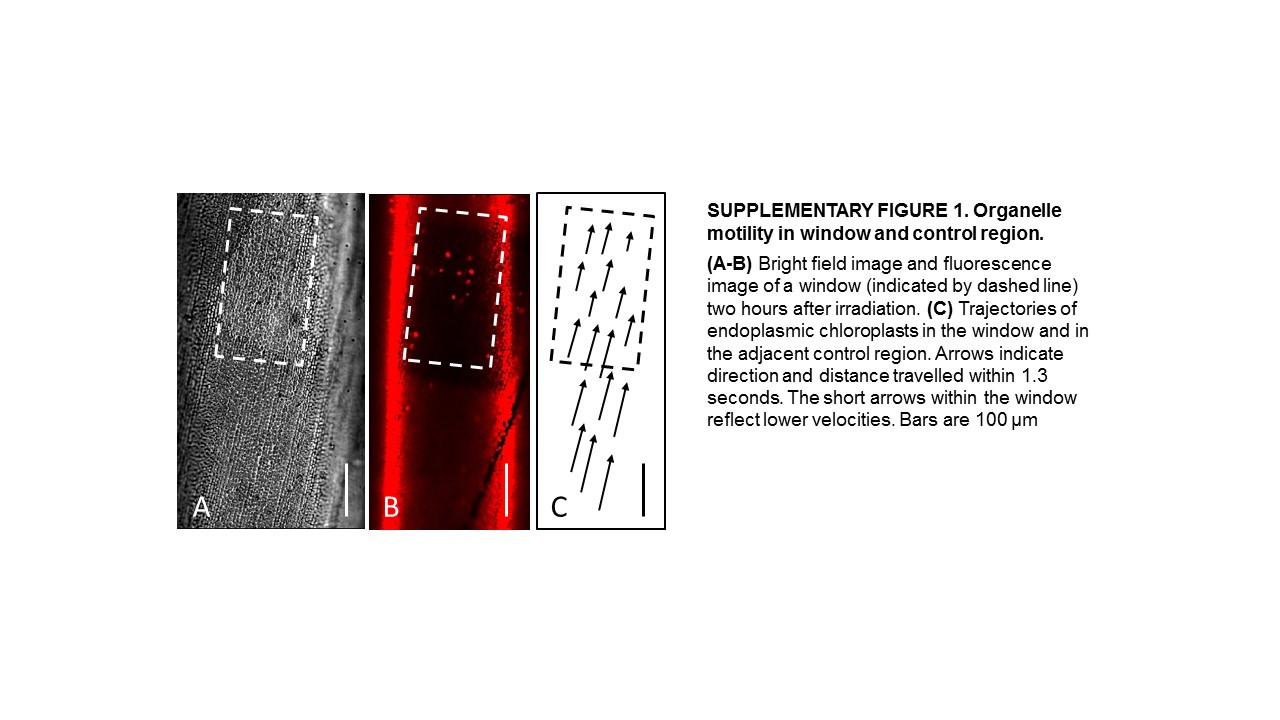

Supplement: Supplementary Figure 1 — Organelle motility in window and control region. (A, B) Bright field image and fluorescence image of a window (indicated by dashed line) two hours after irradiation. (C) Trajectories of endoplasmic (non-bleached) chloroplasts in the window and in the adjacent control region. Arrows indicate direction and distance travelled within 1.3 seconds. The short arrows within the window reflect lower velocities. Bars are 100 µm [file Image1.jpeg]

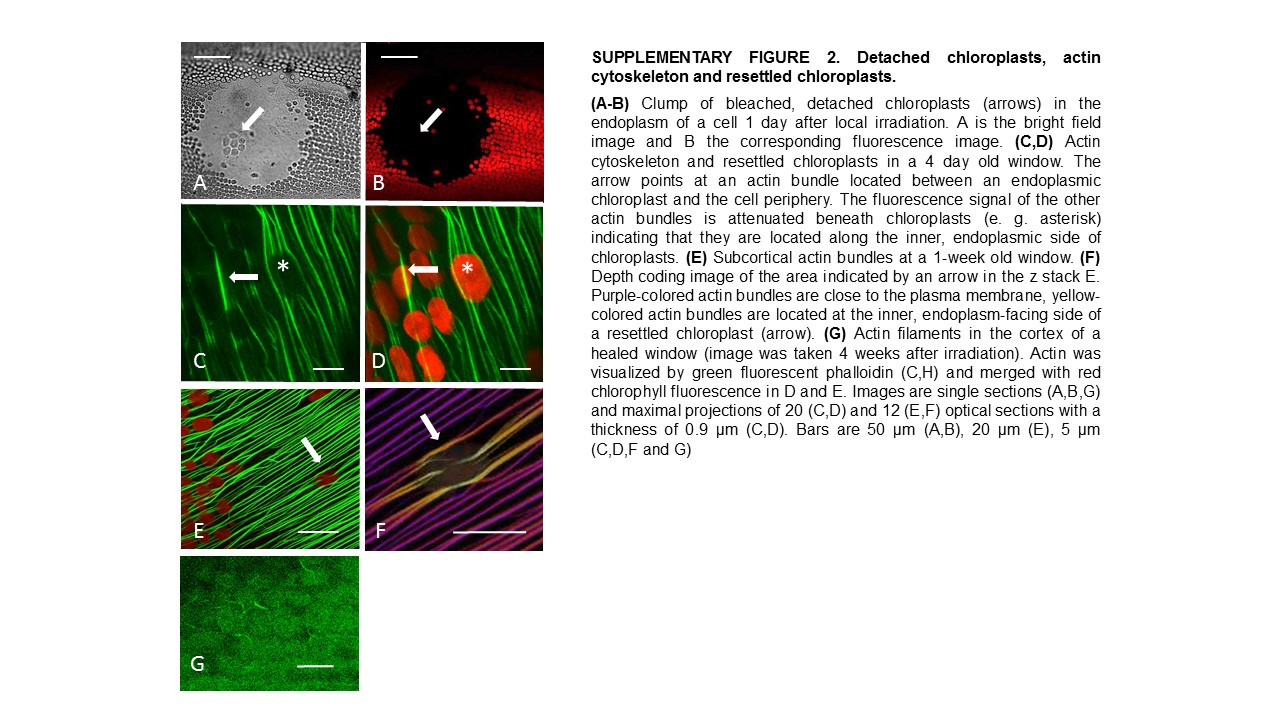

Supplement: Supplementary Figure 2 — Detached chloroplasts, actin cytoskeleton and resettled chloroplasts. (A, B) Clump of bleached, detached chloroplasts (arrows) in the endoplasm of a cell 1 day after local irradiation. A is the bright field image and B the corresponding fluorescence image. (C, D) Actin cytoskeleton and resettled chloroplasts in a 4 day old window. The arrow points at an actin bundle located between an endoplasmic chloroplast and the cell periphery. The fluorescence signal of the other actin bundles is attenuated beneath chloroplasts (e. g. asterisk) indicating that they are located along the inner, endoplasmic side of chloroplasts. (E) Subcortical actin bundles at a 1-week old window. (F) Depth coding image of the area indicated by an arrow in the z stack E. Purple-coloured actin bundles are close to the plasma membrane, yellow-coloured actin bundles are located at the inner, endoplasm-facing side of a resettled chloroplast (arrow). (G) Actin filaments in the cortex of a replenished window (image was taken 4 weeks after irradiation). Actin was visualized by green fluorescent phalloidin (C, H) and merged with red chlorophyll fluorescence in D and E. Images are single sections (A, B, G) and maximum projections of 20 (C, D) and 12 (E) optical sections with a thickness of 0.9 µm (C, D). Bars are 50 µm (A, B), 20 µm (E), 5 µm (C, D, F, G). [file Image2.jpeg]

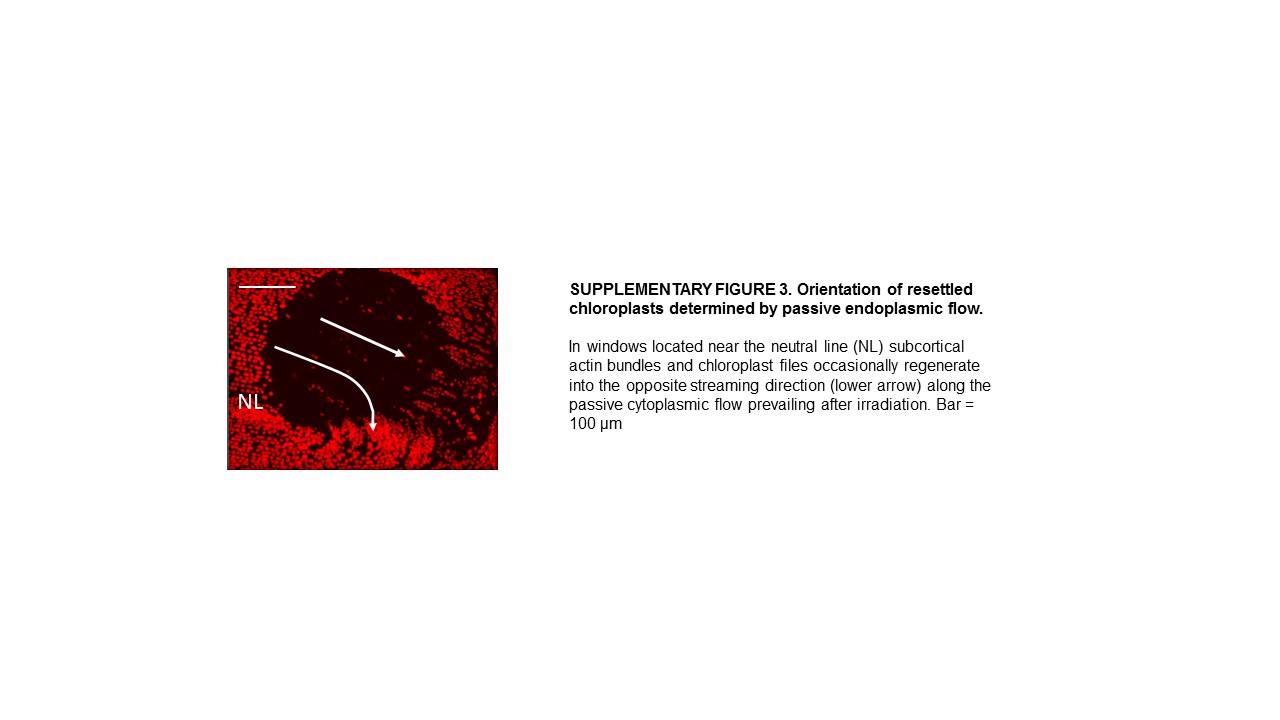

Supplement: Supplementary Figure 3 — Orientation of resettled chloroplasts determined by passive endoplasmic flow. In windows located near the neutral line (NL) subcortical actin bundles and chloroplast files occasionally regenerate into the opposite streaming direction (lower arrow) along the passive cytoplasmic flow prevailing after irradiation. Image is a maximum projection of 15 optical sections with a thickness of 1.3 µm. Bar = 100 µm [file Image3.jpeg]

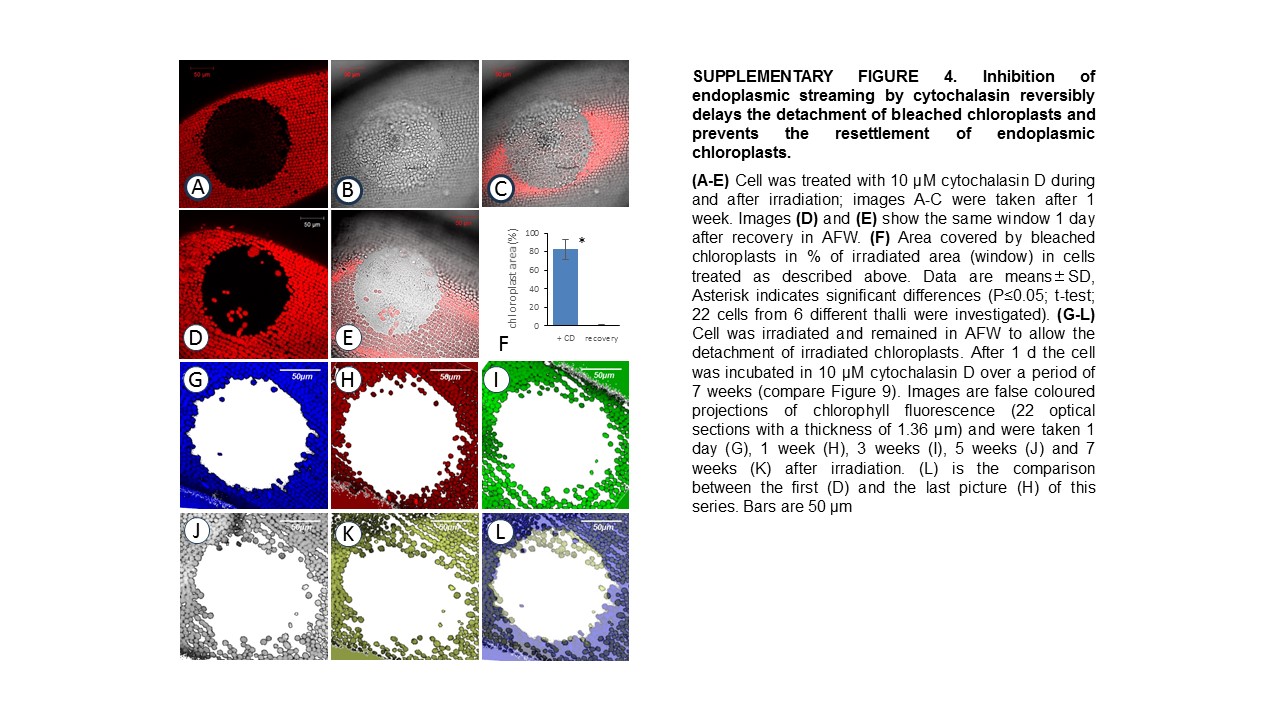

Supplement: Supplementary Figure 4 — Inhibition of endoplasmic streaming by cytochalasin reversibly delays the detachment of bleached chloroplasts and prevents the resettlement of endoplasmic chloroplasts. (A-E) Cell was treated with 10 µM cytochalasin D during and after irradiation; images A-C were taken 1 week after irradiation. Images D and E show the same window 1 day after recovery in AFW. (F) Area covered by bleached chloroplasts in % of irradiated area (window) in cells treated as described above. Data are means±SD, Asterisk indicates significant differences (P≤0.05; t-test; 22 cells from 6 different thalli were investigated). (G-L) Cell was irradiated and remained in AFW to allow the detachment of irradiated chloroplasts. After 1 d the cell was incubated in 10 µM cytochalasin D over a period of 7 weeks (compare Figure 9 ). Images A and D are maximum projections of 5 and 7 optical sections with a thickness of 1.3 µm; images B,C and E are single sections. Images G-L are false coloured projections of chlorophyll fluorescence (22 optical sections with a thickness of 1.36 µm) and were taken 1 day (G), 1 week (H), 3 weeks (I), 5 weeks (J) and 7 weeks (K) after irradiation. (L) is the comparison between the first (D) and the last picture (H) of this series. Bars are 50 µm. [file Image4.jpeg]

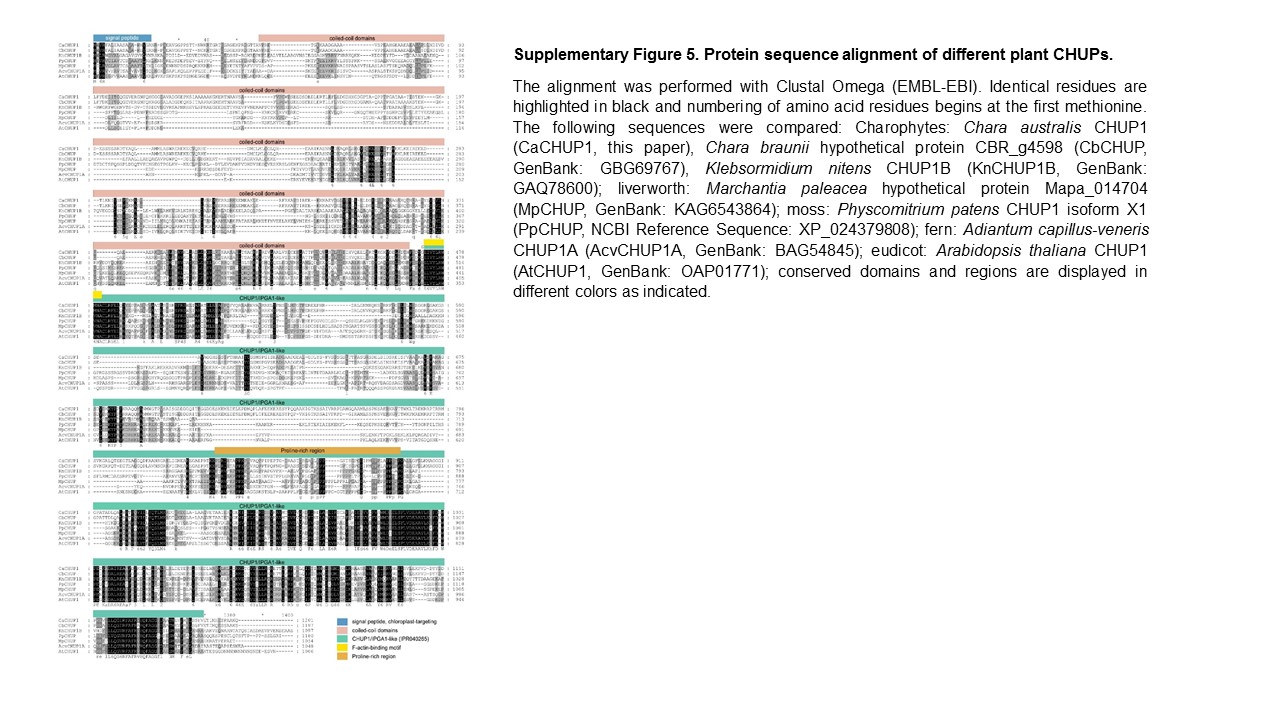

Supplement: Supplementary Figure 5 — Protein sequence alignment of different plant CHUPs. The alignment was performed with Clustal Omega (EMBL-EBI). Identical residues are highlighted in black, and numbering of amino acid residues begins at the first methionine. The following sequences were compared: Charophytes: Chara australis CHUP1 (CaCHUP1, this paper), Chara braunii hypothetical protein CBR_g4598 (CbCHUP, GenBank: GBG69767), Klebsormidium nitens CHUP1B (KnCHUP1B, GenBank: GAQ78600); liverworth: Marchantia paleacea hypothetical protein Mapa_014704 (MpCHUP, GenBank: KAG6543864); moss: Physcomitrium patens CHUP1 isoform X1 (PpCHUP, NCBI Reference Sequence: XP_024379808); fern: Adiantum capillus-veneris CHUP1A (AcvCHUP1A, GenBank: BAG54845); eudicot: Arabidopsis thaliana CHUP1 (AtCHUP1, GenBank: OAP01771); conserved domains and regions are displayed in different colours as indicated. [file Image5.jpeg]
