# Supplementary material for: Recovery of the cortical chloroplast layer in the green alga Chara after local irradiation
Source: Front Plant Sci. 2025 May 5;16:1544999. doi: 10.3389/fpls.2025.1544999 (PMC12086157; doi:10.3389/fpls.2025.1544999)
Supplement: Supplementary Data Sheet 1 — Statistics results of inhibitor experiments with cytochalasin, oryzalin, paclitaxel. [file DataSheet1.pdf]

Inhibitor cytochalasin D; treatment vs control along period of 6 weeks

Mixed Model

Model Info

Info	
Estimate	Linear mixed model fit by REML
Call	window ~ 1 + group + time + group:time+(1 | specimen)
AIC	542.497
BIC	510.851
LogLikel.	−226.098
R-squared Marginal	0.665
R-squared Conditional	0.868
Converged	yes
Optimizer	bobyqa

[3]

Model Results

Fixed Effect Omnibus tests

	F	Num df	Den df	p
group	16.4	1	9.00	0.003
time	13.4	5	45.00	< .001
group ✻ time	15.7	5	45.00	< .001

Note. Satterthwaite method for degrees of freedom

Inhibitor cytochalasin D; treatment vs control along period of 6 weeks

Post Hoc Tests

Post Hoc Comparisons - group

Comparison							
group		group	Difference	SE	t	df	$p_{bonferroni}$
control	-	cytochalasin	−34.7	8.58	−4.05	9.00	0.003

Post Hoc Comparisons - time

Comparison							
time		time	Difference	SE	t	df	$p_{bonferroni}$
1	-	2	4.17	4.65	0.896	45.0	1.000
1	-	3	11.20	4.65	2.408	45.0	0.303
1	-	4	18.68	4.65	4.016	45.0	0.003
1	-	5	24.87	4.65	5.345	45.0	< .001
1	-	6	31.07	4.65	6.678	45.0	< .001
2	-	3	7.03	4.65	1.512	45.0	1.000
2	-	4	14.52	4.65	3.121	45.0	0.047
2	-	5	20.70	4.65	4.450	45.0	< .001
2	-	6	26.90	4.65	5.782	45.0	< .001
3	-	4	7.48	4.65	1.609	45.0	1.000
3	-	5	13.67	4.65	2.938	45.0	0.078
3	-	6	19.87	4.65	4.271	45.0	0.001
4	-	5	6.18	4.65	1.329	45.0	1.000
4	-	6	12.38	4.65	2.662	45.0	0.161
5	-	6	6.20	4.65	1.333	45.0	1.000

Inhibitor oryzalin; treatment vs control after 4 weeks

Independent Samples T-test

Independent Samples T-Test

		Statistic	df	p
oryzalin	Student's t	−0.811	29.0	0.424
	Welch's t	−0.883	28.8	0.385
	Mann-Whitney U	98.0		0.535

Note. H_a $\mu_{control} \neq \mu_{oryzalin}$

Assumptions

Normality Test (Shapiro-Wilk)

	W	p
oryzalin	0.903	0.008

Note. A low p-value suggests a violation of the assumption of normality

Homogeneity of Variances Test (Levene's)

	F	df	df2	p
oryzalin	1.05	1	29	0.313

Note. A low p-value suggests a violation of the assumption of equal variances

Inhibitor paclitaxel; treatment vs control after 6 weeks

Independent Samples T-test

Independent Samples T-Test

		Statistic	df	p
paclitaxel	Student's t	1.33	15.0	0.204
	Welch's t	1.31	13.9	0.210
	Mann-Whitney U	19.0		0.114

Note. H_a $\mu_{control} \neq \mu_{paclitaxel}$

Assumptions

Normality Test (Shapiro-Wilk)

	W	p
paclitaxel	0.887	0.042

Note. A low p-value suggests a violation of the assumption of normality

Homogeneity of Variances Test (Levene's)

	F	df	df2	p
paclitaxel	0.00603	1	15	0.939

Note. A low p-value suggests a violation of the assumption of equal variances
