# Supplementary material for: Recovery of the cortical chloroplast layer in the green alga Chara after local irradiation
Source: Front Plant Sci. 2025 May 5;16:1544999. doi: 10.3389/fpls.2025.1544999 (PMC12086157; doi:10.3389/fpls.2025.1544999)
Supplement: Supplementary Data Sheet 2 — Statistics results of PAM fluorescence measurements control regions and border chloroplasts. [file DataSheet2.pdf]

Window border chloroplasts comparison of Fv/Fm in the dark

Mixed Model

Info	
Model Info	
Estimate	Linear mixed model fit by REML
Call	Fv/Fm ~ 1 + locus + time + locus:time+(1 | cell)
AIC	-1880.022
BIC	-1286.544
LogLikel.	831.201
R-squared Marginal	0.785
R-squared Conditional	0.810
Converged	yes
Optimizer	bobyqa

Model Results

	F	Num df	Den df	p
Fixed Effect Omnibus tests				
**locus**	602.20	5	753	<.001
**time**	11.25	8	754	<.001
**locus ✻ time**	4.10	40	753	<.001

Window border chloroplasts comparison of Fv/Fm in the dark continued (up = upstream, down = downstream, far = distant to the window, close = close to the window, bord = directly at the border of the window)

Post Hoc Tests

Comparison								
locus		locus	Difference	SE	t	df	$p_{bonferroni}$	p_{holm}
Post Hoc Comparisons - locus								
down_bord	-	down_close	-0.29610	0.00892	-33.184	753	<.001	<.001
down_bord	-	down_far	-0.30690	0.00892	-34.394	753	<.001	<.001
down_close	-	down_far	-0.01079	0.00892	-1.209	753	1.000	1.000
up-close	-	down_bord	0.28969	0.00892	32.466	753	<.001	<.001
up-close	-	down_close	-0.00641	0.00892	-0.719	753	1.000	1.000
up-close	-	down_far	-0.01720	0.00892	-1.928	753	0.813	0.380
up-close	-	up_bord	0.29145	0.00892	32.662	753	<.001	<.001
up_bord	-	down_bord	-0.00176	0.00892	-0.197	753	1.000	1.000
up_bord	-	down_close	-0.29786	0.00892	-33.381	753	<.001	<.001
up_bord	-	down_far	-0.30865	0.00892	-34.591	753	<.001	<.001
up_far	-	down_bord	0.30229	0.00892	33.878	753	<.001	<.001
up_far	-	down_close	0.00619	0.00892	0.693	753	1.000	1.000
up_far	-	down_far	-0.00461	0.00892	-0.516	753	1.000	1.000
up_far	-	up-close	0.01260	0.00892	1.412	753	1.000	0.950
up_far	-	up_bord	0.30405	0.00892	34.074	753	<.001	<.001

Window border chloroplasts comparison of Fv/Fm in the dark continued (up = upstream, down = downstream, far = distant to the window, close = close to the window, bord = directly at the border of the window)

Comparison								
time		time	Difference	SE	t	df	$p_{bonferroni}$	p_{holm}
Post Hoc Comparisons - time								
0	-	1	-0.01753	0.0105	-1.6625	753	1.000	1.000
0	-	10	0.02499	0.0105	2.3698	753	0.650	0.307
0	-	17	0.03193	0.0105	3.0282	753	0.092	0.048
0	-	2	-0.01718	0.0105	-1.6295	753	1.000	1.000
0	-	29	0.03924	0.0105	3.7217	753	0.008	0.005
0	-	3	-0.02309	0.0122	-1.8957	758	1.000	0.817
0	-	7	0.01576	0.0105	1.4941	753	1.000	1.000
0	-	79	0.04608	0.0107	4.2912	754	<.001	<.001
1	-	10	0.04252	0.0105	4.0324	753	0.002	0.002
1	-	17	0.04946	0.0105	4.6908	753	<.001	<.001
1	-	2	3.49e-4	0.0105	0.0331	753	1.000	1.000
1	-	29	0.05678	0.0105	5.3843	753	<.001	<.001
1	-	3	-0.00556	0.0122	-0.4562	758	1.000	1.000
1	-	7	0.03329	0.0105	3.1567	753	0.060	0.035
1	-	79	0.06361	0.0107	5.9238	754	<.001	<.001
10	-	17	0.00694	0.0105	0.6584	753	1.000	1.000
10	-	29	0.01426	0.0105	1.3519	753	1.000	1.000
10	-	79	0.02109	0.0107	1.9642	754	1.000	0.748
17	-	29	0.00731	0.0105	0.6935	753	1.000	1.000
17	-	79	0.01415	0.0107	1.3176	754	1.000	1.000
2	-	10	0.04217	0.0105	3.9993	753	0.003	0.002
2	-	17	0.04911	0.0105	4.6577	753	<.001	<.001
2	-	29	0.05643	0.0105	5.3512	753	<.001	<.001
2	-	3	-0.00590	0.0122	-0.4848	758	1.000	1.000
2	-	7	0.03294	0.0105	3.1236	753	0.067	0.037
2	-	79	0.06326	0.0107	5.8913	754	<.001	<.001
29	-	79	0.00684	0.0107	0.6367	754	1.000	1.000
3	-	10	0.04808	0.0122	3.9476	758	0.003	0.002
3	-	17	0.05502	0.0122	4.5177	758	<.001	<.001
3	-	29	0.06233	0.0122	5.1181	758	<.001	<.001
3	-	7	0.03884	0.0122	3.1894	758	0.053	0.033
3	-	79	0.06917	0.0124	5.5920	759	<.001	<.001
7	-	10	0.00923	0.0105	0.8757	753	1.000	1.000
7	-	17	0.01618	0.0105	1.5341	753	1.000	1.000
7	-	29	0.02349	0.0105	2.2276	753	0.943	0.419
7	-	79	0.03033	0.0107	2.8241	754	0.175	0.088

Window border chloroplasts comparison of Fv'/Fm' at low light L20

Mixed Model

Info	
Model Info	
Estimate	Linear mixed model fit by REML
Call	L20 ~ 1 + locus + time + locus:time+(1 | cell)
AIC	-1954.079
BIC	-1355.718
LogLikel.	865.788
R-squared Marginal	0.767
R-squared Conditional	0.806
Converged	yes
Optimizer	bobyqa

Model Results

	F	Num df	Den df	p
Fixed Effect Omnibus tests				
**locus**	579.83	5	753	<.001
**time**	12.82	8	754	<.001
**locus ∗ time**	3.36	40	753	<.001

Window border chloroplasts comparison of Fv'/Fm' at low light L20 continued (up = upstream, down = downstream, far = distant to the window, close = close to the window, bord = directly at the border of the window)

Post Hoc Tests

Comparison								
locus		locus	Difference	SE	t	df	$p_{bonferroni}$	p_{holm}
Post Hoc Comparisons - locus								
down_bord	-	down_close	-0.27498	0.00850	-32.360	753	<.001	<.001
down_bord	-	down_far	-0.28853	0.00850	-33.955	753	<.001	<.001
down_close	-	down_far	-0.01355	0.00850	-1.595	753	1.000	0.556
up-close	-	down_bord	0.26504	0.00850	31.190	753	<.001	<.001
up-close	-	down_close	-0.00994	0.00850	-1.170	753	1.000	0.970
up-close	-	down_far	-0.02350	0.00850	-2.765	753	0.087	0.041
up-close	-	up_bord	0.26941	0.00850	31.705	753	<.001	<.001
up_bord	-	down_bord	-0.00438	0.00850	-0.515	753	1.000	1.000
up_bord	-	down_close	-0.27935	0.00850	-32.875	753	<.001	<.001
up_bord	-	down_far	-0.29291	0.00850	-34.470	753	<.001	<.001
up_far	-	down_bord	0.28177	0.00850	33.159	753	<.001	<.001
up_far	-	down_close	0.00679	0.00850	0.800	753	1.000	1.000
up_far	-	down_far	-0.00676	0.00850	-0.795	753	1.000	1.000
up_far	-	up-close	0.01674	0.00850	1.969	753	0.739	0.296
up_far	-	up_bord	0.28615	0.00850	33.674	753	<.001	<.001

Window border chloroplasts comparison of Fv'/Fm' at low light L20 continued (up = upstream, down = downstream, far = distant to the window, close = close to the window, bord = directly at the border of the window)

Comparison								
time		time	Difference	SE	t	df	$p_{bonferroni}$	p_{holm}
Post Hoc Comparisons - time								
0	-	1	-0.03740	0.0100	-3.7239	753	0.008	0.005
0	-	10	9.90e-4	0.0100	0.0985	753	1.000	1.000
0	-	17	0.00805	0.0100	0.8018	753	1.000	1.000
0	-	2	-0.04476	0.0100	-4.4573	753	<.001	<.001
0	-	29	0.01340	0.0100	1.3340	753	1.000	1.000
0	-	3	-0.05712	0.0116	-4.9229	757	<.001	<.001
0	-	7	-0.01658	0.0100	-1.6509	753	1.000	1.000
0	-	79	0.01857	0.0102	1.8157	754	1.000	1.000
1	-	10	0.03839	0.0100	3.8225	753	0.005	0.003
1	-	17	0.04545	0.0100	4.5258	753	<.001	<.001
1	-	2	-0.00736	0.0100	-0.7334	753	1.000	1.000
1	-	29	0.05079	0.0100	5.0579	753	<.001	<.001
1	-	3	-0.01973	0.0116	-1.7001	757	1.000	1.000
1	-	7	0.02082	0.0100	2.0731	753	1.000	0.616
1	-	79	0.05597	0.0102	5.4722	754	<.001	<.001
10	-	17	0.00706	0.0100	0.7033	753	1.000	1.000
10	-	29	0.01241	0.0100	1.2354	753	1.000	1.000
10	-	79	0.01758	0.0102	1.7189	754	1.000	1.000
17	-	29	0.00534	0.0100	0.5321	753	1.000	1.000
17	-	79	0.01052	0.0102	1.0284	754	1.000	1.000
2	-	10	0.04575	0.0100	4.5558	753	<.001	<.001
2	-	17	0.05281	0.0100	5.2591	753	<.001	<.001
2	-	29	0.05816	0.0100	5.7913	753	<.001	<.001
2	-	3	-0.01236	0.0116	-1.0654	757	1.000	1.000
2	-	7	0.02818	0.0100	2.8064	753	0.185	0.092
2	-	79	0.06333	0.0102	6.1922	754	<.001	<.001
29	-	79	0.00517	0.0102	0.5059	754	1.000	1.000
3	-	10	0.05811	0.0116	5.0082	757	<.001	<.001
3	-	17	0.06517	0.0116	5.6169	757	<.001	<.001
3	-	29	0.07052	0.0116	6.0774	757	<.001	<.001
3	-	7	0.04054	0.0116	3.4942	757	0.018	0.011
3	-	79	0.07569	0.0118	6.4220	758	<.001	<.001
7	-	10	0.01757	0.0100	1.7494	753	1.000	1.000
7	-	17	0.02463	0.0100	2.4527	753	0.519	0.245
7	-	29	0.02997	0.0100	2.9848	753	0.105	0.056
7	-	79	0.03515	0.0102	3.4366	754	0.022	0.012

Window border chloroplasts comparison of Fv'/Fm' at high light L60

Mixed Model

Info	
Model Info	
Estimate	Linear mixed model fit by REML
Call	L60 ~ 1 + locus + time + locus:time+(1 | cell)
AIC	-2212.121
BIC	-1596.780
LogLikel.	986.319
R-squared Marginal	0.633
R-squared Conditional	0.735
Converged	yes
Optimizer	bobyqa

Model Results

	F	Num df	Den df	p
Fixed Effect Omnibus tests				
**locus**	319.12	5	753	<.001
**time**	23.64	8	753	<.001
**locus ✻ time**	3.01	40	753	<.001

Window border chloroplasts comparison of Fv'/Fm' at high light L60 continued (up = upstream, down = downstream, far = distant to the window, close = close to the window, bord = directly at the border of the window)

Post Hoc Tests

Comparison								
locus		locus	Difference	SE	t	df	$p_{bonferroni}$	p_{holm}
Post Hoc Comparisons - locus								
down_bord	-	down_close	-0.16749	0.00722	-23.197	753	<.001	<.001
down_bord	-	down_far	-0.18077	0.00722	-25.036	753	<.001	<.001
down_close	-	down_far	-0.01328	0.00722	-1.839	753	0.995	0.265
up-close	-	down_bord	0.16256	0.00722	22.514	753	<.001	<.001
up-close	-	down_close	-0.00493	0.00722	-0.683	753	1.000	0.990
up-close	-	down_far	-0.01821	0.00722	-2.522	753	0.178	0.059
up-close	-	up_bord	0.16469	0.00722	22.809	753	<.001	<.001
up_bord	-	down_bord	-0.00213	0.00722	-0.295	753	1.000	0.990
up_bord	-	down_close	-0.16962	0.00722	-23.492	753	<.001	<.001
up_bord	-	down_far	-0.18290	0.00722	-25.331	753	<.001	<.001
up_far	-	down_bord	0.18789	0.00722	26.022	753	<.001	<.001
up_far	-	down_close	0.02040	0.00722	2.825	753	0.073	0.029
up_far	-	down_far	0.00712	0.00722	0.986	753	1.000	0.973
up_far	-	up-close	0.02533	0.00722	3.508	753	0.007	0.003
up_far	-	up_bord	0.19002	0.00722	26.317	753	<.001	<.001

Window border chloroplasts comparison of Fv’/Fm’ at high light L60 continued (up = upstream, down = downstream, far = distant to the window, close = close to the window, bord = directly at the border of the window)

Comparison								
time		time	Difference	SE	t	df	$p_{bonferroni}$	p_{holm}
Post Hoc Comparisons - time								
0	-	1	-0.02895	0.00853	-3.393	753	0.026	0.011
0	-	10	-0.03517	0.00853	-4.121	753	0.002	<.001
0	-	17	-0.03912	0.00853	-4.585	753	<.001	<.001
0	-	2	-0.07853	0.00853	-9.203	753	<.001	<.001
0	-	29	-0.04092	0.00853	-4.796	753	<.001	<.001
0	-	3	-0.11489	0.00986	-11.647	755	<.001	<.001
0	-	7	-0.06593	0.00853	-7.727	753	<.001	<.001
0	-	79	-0.04543	0.00869	-5.227	753	<.001	<.001
1	-	10	-0.00621	0.00853	-0.728	753	1.000	1.000
1	-	17	-0.01017	0.00853	-1.191	753	1.000	1.000
1	-	2	-0.04958	0.00853	-5.810	753	<.001	<.001
1	-	29	-0.01197	0.00853	-1.403	753	1.000	1.000
1	-	3	-0.08593	0.00986	-8.712	755	<.001	<.001
1	-	7	-0.03698	0.00853	-4.334	753	<.001	<.001
1	-	79	-0.01648	0.00869	-1.896	753	1.000	0.642
10	-	17	-0.00395	0.00853	-0.463	753	1.000	1.000
10	-	29	-0.00576	0.00853	-0.674	753	1.000	1.000
10	-	79	-0.01026	0.00869	-1.181	753	1.000	1.000
17	-	29	-0.00180	0.00853	-0.211	753	1.000	1.000
17	-	79	-0.00631	0.00869	-0.726	753	1.000	1.000
2	-	10	0.04336	0.00853	5.082	753	<.001	<.001
2	-	17	0.03941	0.00853	4.619	753	<.001	<.001
2	-	29	0.03761	0.00853	4.408	753	<.001	<.001
2	-	3	-0.03636	0.00986	-3.686	755	0.009	0.004
2	-	7	0.01260	0.00853	1.477	753	1.000	1.000
2	-	79	0.03310	0.00869	3.809	753	0.005	0.003
29	-	79	-0.00451	0.00869	-0.519	753	1.000	1.000
3	-	10	0.07972	0.00986	8.082	755	<.001	<.001
3	-	17	0.07577	0.00986	7.681	755	<.001	<.001
3	-	29	0.07396	0.00986	7.499	755	<.001	<.001
3	-	7	0.04895	0.00986	4.963	755	<.001	<.001
3	-	79	0.06946	0.01002	6.932	756	<.001	<.001
7	-	10	0.03077	0.00853	3.606	753	0.012	0.005
7	-	17	0.02681	0.00853	3.142	753	0.063	0.024
7	-	29	0.02501	0.00853	2.931	753	0.125	0.045
7	-	79	0.02050	0.00869	2.359	753	0.668	0.223
