# Supplementary material for: Recovery of the cortical chloroplast layer in the green alga Chara after local irradiation
Source: Front Plant Sci. 2025 May 5;16:1544999. doi: 10.3389/fpls.2025.1544999 (PMC12086157; doi:10.3389/fpls.2025.1544999)
Supplement: Supplementary Data Sheet 3 — Statistics results of PAM fluorescence measurements control regions and resettled chloroplasts. [file DataSheet3.pdf]

Resettled chloroplasts comparison of Fv/Fm in the dark (up_far = upstream to the window, win = window, down_far = downstream)

Mixed Model

Info	
Model Info	
Estimate	Linear mixed model fit by REML
Call	Fv/Fm ~ 1 + locus + time + locus:time+(1 | cell)
AIC	-1087.768
BIC	-848.172
LogLikel.	489.907
R-squared Marginal	0.143
R-squared Conditional	0.444
Converged	yes
Optimizer	bobyqa

Model Results

	F	Num df	Den df	p
Fixed Effect Omnibus tests				
**locus**	28.661	2	270	<.001
**time**	1.923	6	271	0.077
**locus ∗ time**	0.882	12	270	0.566

Post Hoc Tests

Comparison							
locus		locus	Difference	SE	t	df	$p_{bonferroni}$
Post Hoc Comparisons - locus							
up_far	-	down_far	-0.00439	0.00516	-0.850	270	1.000
up_far	-	wind	0.03372	0.00543	6.214	270	<.001
wind	-	down_far	-0.03811	0.00543	-7.022	270	<.001

Resettled chloroplasts comparison of Fv'/Fm' at low light L20 (up_far = upstream to the window, win = window, down_far = downstream)

Mixed Model

Info	
**Model Info**	
Estimate	Linear mixed model fit by REML
Call	L20 ~ 1 + locus + time + locus:time+(1 | cell)
AIC	-1028.966
BIC	-793.401
LogLikel.	462.522
R-squared Marginal	0.193
R-squared Conditional	0.487
Converged	yes
Optimizer	bobyqa

Model Results

	F	Num df	Den df	p
Fixed Effect Omnibus tests				
**locus**	33.42	2	270	<.001
**time**	3.95	6	271	<.001
**locus ✻ time**	1.91	12	270	0.033

Post Hoc Tests

Comparison							
locus		locus	Difference	SE	t	df	$p_{bonferroni}$
Post Hoc Comparisons - locus							
up_far	-	down_far	-0.00642	0.00567	-1.13	270	0.776
up_far	-	wind	0.03928	0.00597	6.58	270	<.001
wind	-	down_far	-0.04570	0.00597	-7.66	270	<.001

Resettled chloroplasts comparison of Fv'/Fm' at low light L20 continued (up_far = upstream to the window, win = window, down_far = downstream)

Comparison							
time		time	Difference	SE	t	df	$p_{bonferroni}$
Post Hoc Comparisons - time							
10	-	17	-0.00109	0.00828	-0.1317	270	1.000
10	-	29	-0.00955	0.00828	-1.1531	270	1.000
10	-	79	0.00409	0.00844	0.4850	270	1.000
17	-	29	-0.00846	0.00828	-1.0214	270	1.000
17	-	79	0.00518	0.00844	0.6142	270	1.000
2	-	10	0.01761	0.00860	2.0464	270	0.875
2	-	17	0.01652	0.00860	1.9196	270	1.000
2	-	29	0.00806	0.00860	0.9366	270	1.000
2	-	3	-0.00905	0.01055	-0.8571	272	1.000
2	-	7	-0.00868	0.00860	-1.0086	270	1.000
2	-	79	0.02170	0.00876	2.4764	270	0.292
29	-	79	0.01364	0.00844	1.6165	270	1.000
3	-	10	0.02665	0.01026	2.5985	272	0.207
3	-	17	0.02556	0.01026	2.4922	272	0.279
3	-	29	0.01711	0.01026	1.6676	272	1.000
3	-	7	3.69e-4	0.01026	0.0360	272	1.000
3	-	79	0.03075	0.01042	2.9502	272	0.072
7	-	10	0.02628	0.00828	3.1742	270	0.035
7	-	17	0.02519	0.00828	3.0425	270	0.054
7	-	29	0.01674	0.00828	2.0211	270	0.929
7	-	79	0.03038	0.00844	3.5996	270	0.008

Resettled chloroplasts comparison of Fv'/Fm' at high light L60 (up_far = upstream to the window, win = window, down_far = downstream)

Mixed Model

Info

Model Info	
Estimate	Linear mixed model fit by REML
Call	L60 ~ 1 + locus + time + locus:time+(1 | cell)
AIC	-915.879
BIC	-688.065
LogLikel.	409.854
R-squared Marginal	0.142
R-squared Conditional	0.480
Converged	yes
Optimizer	bobyqa

Model Results

Fixed Effect Omnibus tests

	F	Num df	Den df	p
**locus**	9.82	2	270	<.001
**time**	7.20	6	271	<.001
**locus ∗ time**	1.18	12	270	0.297

Post Hoc Tests

Post Hoc Comparisons - locus

Comparison locus		locus	Difference	SE	t	df	$p_{bonferroni}$
up_far	-	down_far	0.00753	0.00680	1.11	270	0.808
up_far	-	wind	0.03078	0.00716	4.30	270	<.001
wind	-	down_far	-0.02325	0.00716	-3.25	270	0.004

Resettled chloroplasts comparison of Fv'/Fm' at high light L60 continued (up_far = upstream to the window, win = window, down_far = downstream)

Comparison							
time		time	Difference	SE	t	df	$P_{bonferroni}$
Post Hoc Comparisons - time							
10	-	17	0.00235	0.00993	0.237	270	1.000
10	-	29	-0.01409	0.00993	-1.419	270	1.000
10	-	79	-0.01531	0.01012	-1.513	270	1.000
17	-	29	-0.01644	0.00993	-1.656	270	1.000
17	-	79	-0.01767	0.01012	-1.745	270	1.000
2	-	10	0.01977	0.01032	1.916	270	1.000
2	-	17	0.02213	0.01032	2.144	270	0.692
2	-	29	0.00568	0.01032	0.550	270	1.000
2	-	3	-0.04325	0.01266	-3.416	272	0.015
2	-	7	-0.01792	0.01032	-1.736	270	1.000
2	-	79	0.00446	0.01051	0.424	270	1.000
29	-	79	-0.00122	0.01012	-0.121	270	1.000
3	-	10	0.06303	0.01230	5.122	272	<.001
3	-	17	0.06538	0.01230	5.313	272	<.001
3	-	29	0.04893	0.01230	3.977	272	0.002
3	-	7	0.02533	0.01230	2.059	272	0.850
3	-	79	0.04771	0.01250	3.816	272	0.004
7	-	10	0.03769	0.00993	3.795	270	0.004
7	-	17	0.04005	0.00993	4.032	270	0.002
7	-	29	0.02360	0.00993	2.376	270	0.382
7	-	79	0.02238	0.01012	2.211	270	0.586
